# Integrative taxonomy and preliminary assessment of species limits in the *Liolaemus walkeri* complex (Squamata, Liolaemidae) with descriptions of three new species from Peru

**DOI:** 10.3897/zookeys.364.6109

**Published:** 2013-12-18

**Authors:** César Aguilar, Perry L. Wood Jr, Juan C. Cusi, Alfredo Guzmán, Frank Huari, Mikael Lundberg, Emma Mortensen, César Ramírez, Daniel Robles, Juana Suárez, Andres Ticona, Víctor J. Vargas, Pablo J. Venegas, Jack W. Sites Jr

**Affiliations:** 1 Department of Biology and Bean Life Science Museum, Brigham Young University (BYU), Provo, UT 84602, USA; 2 Departamento de Herpetología, Museo de Historia Natural de San Marcos (MUSM), Av. Arenales 1256, Jesús María, Lima, Peru; 3 Instituto de Ciencias Biológicas Antonio Raimondi, Facultad de Ciencias Biológicas, Universidad Nacional Mayor de San Marcos, Lima, Peru; 4 Asociación Pro Fauna Silvestre, Urb. Mariscal Cáceres Mz. L - Lt. 48, Huamanga, Ayacucho, Peru; 5 División de Herpetología-Centro de Ornitología y Biodiversidad (CORBIDI), Santa Rita N˚105 Of. 202, Urb. Huertos de San Antonio, Surco, Lima, Peru

**Keywords:** *Liolaemus walkeri* complex, integrative taxonomy, new species, viviparity

## Abstract

Species delimitation studies based on integrative taxonomic approaches have received considerable attention in the last few years, and have provided the strongest hypotheses of species boundaries. We used three lines of evidence (molecular, morphological, and niche envelopes) to test for species boundaries in Peruvian populations of the *Liolaemus walkeri* complex. Our results show that different lines of evidence and analyses are congruent in different combinations, for unambiguous delimitation of three lineages that were “hidden” within known species, and now deserve species status. Our phylogenetic analysis shows that *L. walkeri*, *L. tacnae* and the three new species are strongly separated from other species assigned to the *alticolor-bibronii* group. Few conventional morphological characters distinguish the new species from closely related taxa and this highlights the need to integrate other sources of data to erect strong hypothesis of species limits. A taxonomic key for known Peruvian species of the subgenus *Lioalemus* is provided.

## Abstract (es)

Los estudios sobre delimitación de especies basados en un enfoque integral han recibido considerable atención en los últimos años, y proveen las hipótesis más robustas sobre límites de especies. Usamos tres líneas de evidencia (molecular, morfológica y modelos de nichos) para evaluar los límites de especies entre poblaciones peruanas del complejo *Liolaemus walkeri*. Nuestros resultados muestran que las diferentes líneas de evidencia y análisis en diferentes combinaciones son congruentes en el descubrimiento no ambiguo de tres linajes que estuvieron confundidos con especies ya conocidas y que ahora merecen reconocimiento específico. Nuestro análisis filogenético muestra que *L. walkeri*, *L. tacnae* y las tres nuevas especies están bien distanciadas de las otras especies asignadas al grupo *alticolor-bibronii*. Pocos caracteres morfológicos convencionales distinguen las nuevas especies de otras estrechamente relacionadas, y esto indica la necesidad de integración de diferentes fuentes de datos para elaborar hipótesis más sólidas sobre límites entre especies. Se proporciona una clave taxonómica para las especies peruanas conocidas del subgénero *Liolaemus*.

## Introduction

The issue of species delimitation (building explicit hypotheses about species lineages and their geographic boundaries) has received considerable attention in the last decade due in part to an emerging consensus about species concepts and new approaches for testing species boundaries (Sites and Marshall 2003, 2004, de Queiroz 2007, Knowles and Carstens 2007, Wiens 2007, Padial and De la Riva 2010, Padial et al. 2010, Hart 2011, Zapata and Jiménez 2012, Camargo and Sites 2013). The ontological General Lineage Concept (GLC) defines a species as a group of separately evolving meta-population lineages, originally proposed by Mayden (1997, 2002) and de Queiroz (1998, 2005). This definition is generally supported by a consensus view in evolutionary biology (Padial and De la Riva 2010, Padial et al. 2010, Hart 2011, Zapata and Jiménez 2012, but see Hausdorf 2011). The GLC distinguishes the primary property (species are separately evolving meta-population lineages) that is shared by most previous competing species concepts (e.g., biological, phylogenetic, ecological species concept, etc.), from secondary properties (e.g., reproductive isolation, character fixation, niche differentiation, etc.) that arise at different times during the processes of speciation (de Queiroz 2007). These secondary properties are lines of evidence that are relevant to inferring the species boundaries (de Queiroz 2005, 2007).

In addition to this agreement with respect to GLC, there is a growing number of new empirical methods of species delimitation (SDL; Pons et al. 2006, Knowles and Carstens 2007, Kubatko et al. 2009, Carstens and Dewey 2010, Flot et al. 2010, Hausdorf and Hennig 2010, Martínez-Gordillo et al. 2010, Gurgel-Gonçalves et al. 2011). These new methods for testing hypotheses of species boundaries have been accommodated under the new term “integrative taxonomy” (IT; Dayrat 2005, Padial and De la Riva 2010, Padial et al. 2010). Methods such as the multi-locus coalescent to infer species limits without monophyletic lineages, ecological niche modeling (ENM) to assess spatial distributions of closely related species, and multivariate tolerance regions to test for discontinuities or gaps in morphology, have all been used in new integrative taxonomic studies (Omland et al. 2006, Knowles and Carstens 2007, Raxworthy et al. 2007, Rissler and Apodaca 2007, Vasconcelos et al. 2012, Zapata and Jiménez 2012).

Character fixation as well as discontinuities or gaps have been used as a SDL criterion to assess species limits based on genetic and morphological characters (Marshall et al. 2006, Zapata and Jiménez 2012). Fixed differences and gaps in morphology suggest that some evolutionary force (e.g., absence of gene flow, natural selection) prevent two putative taxa from homogenizing (Wiens and Servedio 2000, Zapata and Jiménez 2012). Often analysis of variance or discriminant analysis have been used to evaluate morphological differentiation in SDL studies, but these statistics, even if significant, evaluate central tendencies and not gaps in morphology, and the latter may be more relevant for testing species boundaries (Zapata and Jiménez 2012). In addition to character fixation and gaps in morphology, niche envelopes can be used to assess the status of uncertain populations which are separated from closely related species by areas that are outside of the climatic niche envelope, and where gene flow between these species is unlikely because it would involve crossing unsuitable habitat (Wiens and Graham 2005). Ecological niche modeling (ENM) can summarize niche envelopes and this approach has also been used in SDL studies (e.g., Raxworthy et al. 2007, Rissler and Apodaca 2007).

Well-supported hypotheses of species boundaries are essential because species are used as basic units of analysis in several areas of biogeography, ecology, and macroevolution, and from the broader perspective of evolutionary theory, delimiting species is important in the context of understanding many evolutionary mechanisms and processes (Sites and Marshall 2003, 2004, Wiens 2007). Among animal groups, lizards have been used extensively in evolutionary studies ranging from community ecology, behavioral ecology, multiple origins of body elongation coupled with limb reduction/loss, multiple origins of novel reproductive modes, including parthenogenesis and viviparity (Sites et al. 2011), as well as phylogeography and speciation studies (Camargo et al. 2010).

SDL studies in lizards have included molecular markers, morphological characters and/or models of species distributions (Camargo et al. 2010). In particular, several clades of the genus *Liolaemus* Wiegmann, 1834 have been studied intensively using molecular and morphological data to delimit species and infer phylogeographic histories (Morando et al. 2008, Victoriano et al. 2008, Breitman et al. 2011a, 2012), and for testing hypotheses about evolutionary processes (Olave et al. 2011) and performance (in accuracy and precision) of different SDL methods (Camargo et al. 2012). This South American genus includes ~ 230 species (Breitman et al. 2011b), and extends from central Peru to Tierra del Fuego, and from sea level on both Atlantic and Pacific coasts to almost 5000 m in elevation. Species diversity is highest in the Andes and adjacent arid regions, and new species descriptions are published at a rate of 4–5/yr, from moderately well-known areas in Argentina and Chile.

In most cases these studies have demonstrated that populations assigned to single species based on generalized morphological features and limited field sampling, tend to under-represent biodiversity. Distinct lineages have been revealed by molecular data, many of which are later described as new species (e.g., Breitman et al. 2011a, b). The largest poorly-known areas for the genus are the Andean regions of Bolivia, Peru and northern Chile. Intensive fieldwork and molecular phylogenetic studies have never been systematically carried out in these regions, and species descriptions have traditionally been based on gross comparisons of morphological characters from small sample sizes and limited geographic sampling. So SDL studies are needed in the extreme northern range of *Liolaemus* (e.g., Peru) based on intensive geographic sampling and large series for collection of new molecular, coloration, and various classes of morphological data.

Currently, 14 species of *Liolaemus* are known from Peru (*Liolaemus montanus* group, 10 spp; *Liolaemus alticolor* group, 4 spp), but SDL studies based on an integrative approach have not been carried out in either of these groups. Moreover, several areas in the Peruvian Andes remain completely unexplored, and based on recent studies in the southern range of *Liolaemus*, it is highly probable that the Peruvian Andes harbor many undiscovered species. Here, we use new molecular, morphological, and geographic data from known Peruvian species (*Liolaemus alticolor* Barbour, 1909, *Liolaemus incaicus* Lobo, Quinteros & Gómez, 2007, *Liolaemus tacnae* (Shreve, 1941) and *Liolaemus walkeri* Shreve, 1938), assigned to the *Liolaemus alticolor* group, and three populations morphologically similar to *Liolaemus walkeri* (identified by their regions of occurrence: Ancash, Ayacucho and Cusco), to present the first SDL study based on an IT approach. Our results provide evidence that three new lineages deserve species status, and these are described herein.

## Methods

### Sampling and DNA extraction

Lizards were collected by hand, photographed and sacrificed with an injection of pentobarbital. After liver tissue was collected for DNA samples, whole specimens were fixed in formaldehyde at 10% and transferred to 70% ethanol for permanent storage in museum collections. Tissue samples were collected in duplicate, stored in 96% ethanol and deposited at the Bean Life Science museum at Brigham Young University (BYU) and Museo de Historia Natural de San Marcos (MUSM) (see Data resources below). Total genomic DNA is extracted from liver/muscle tissue following the protocol of Fetzner (1999), and using Qiagen DNeasy kits (Qiagen, Inc., Valencia, CA).

### Mitochondrial DNA amplification and sequencing

Forty-eight samples from 40 localities were sequenced for 669bp of the mtDNA cytochrome b (cyt-b) region, with LIO742F 5’–TCGACCTVCCYGCCCCATCA–3’ and LIO742R 5’–GAGGGGTTACTAAGGGGTTGGC–3’ primers (this study), and all unique cyt-b haplotypes were sequenced for a 12S region (752 bp) using primers 12Stphe 5’AAAGCACRGCACTGAAGATGC–3’ and 12SE 5’–GTRCGCTTACCWTGTTACGACT–3’ (Wiens et al. 1999). Double stranded polymerase chain reactions (PCR) were amplified under the following conditions: 1.0 μL of genomic DNA, 1.0 μL of light strand primer 1.0 of μL of heavy strand primer, 1.0 μL of dinucleotide pairs, 2.0 μL of 5x~ buffer, 1.0 μL of MgCl 10x~ buffer, 0.18 μL of Taq polymerase, and 7.5 μL of diH2O. PCR amplification was executed under the following conditions: initial denaturation at 95°C for 2 min, followed by a second denaturation at 95°C for 35 s, annealing at 52°C for 35 s, followed by a cycle extension at 72°C for 35 s, for 31 cycles. PCR products were visualized on a 10% agarose gels to ensure the targeted products were cleanly amplified, and then purified using a MultiScreen PCR (mu) 96 (Millipore Corp., Billerica, MA) and directly sequenced using the BigDye Terminator v 3.1 Cycle Sequencing Ready Reaction (Applied Biosystems, Foster City, CA). The cycle sequencing reactions were purified using Sephadex G-50 Fine (GE Healthcare) and MultiScreen HV plates (Millipore Corp.). Samples were then analyzed on a ABI3730xl DNA Analyzer in the BYU DNA Sequencing Center.

### Phylogenetic reconstruction

All sequences were aligned in MUSCLE (Edgar 2004) plugin, and cyt-b sequences were translated to check for premature stop codons in GENEIOUS®PRO v5.6.6. Cyt b haplotype diversity was estimated using DnaSP (Librado and Rozas 2009), and concatenated cyt-b and 12S regions were edited using GENEIOUS®PRO (Drummond et al. 2011). For ingroups and outgroups we used selected species of the subgenus *Liolaemus* that are assigned to different species groups and for which cyt-b and 12S sequences are available in GenBank. Our ingroup samples included taxa that have been assigned to the same species group as *Liolaemus tacnae* and *Liolaemus walkeri* (*alticolor-bibronii* group), including: *Liolaemus abdalai* Quinteros, *Liolaemus bibronii* (Bell), *Liolaemus gracilis* (Bell), *Liolaemus ramirezae* Lobo & Espinoza and *Liolaemus saxatilis* Ávila & Cei (Lobo et al. 2010). To further test for monophyly of the *alticolor-bibroni* group, we sampled three species assigned to different species groups (*robertmertensi, pictus* and *monticola* groups), but nested within the subgenus *Liolaemus*; these include: *Liolaemus monticola* Müller & Hellmich, *Liolaemus pictus* Duméril & Bribon and *Liolaemus robertmertensi* Hellmich (Lobo et al. 2010). We used *Liolaemus lineomaculatus* Boulenguer, a species belonging to the subgenus *Eulaemus* (Lobo et al. 2010, Fontanella et al. 2012) as the outgroup. All new sequences were deposited in GenBank (accession numbers KF923633–KF923660 and KF923661–KF923688 for cyt-b and 12s respectively) and a list of all haplotypes, GenBank accession and museum voucher numbers used for the phylogenetic analysis are provided as Supplementary file 1.

Bayesian Information Criteria in JMODELTEST (v 0.01; Posada 2005) identified the best-fit model of evolution for the complete data set of haplotypes as TPM2 + I + Γ. A Maximum-likelihood (ML) search in PHYML (Guindon and Gascuel 2003) was performed with 1000 replicates for bootstrap analyses; we consider strong nodal support for bootstrap values ≥ 70 (Hillis and Bull 1993; with caveats). Because the TPM2 + I + G model is not incorporated in the MRBAYES (Huelsenbeck and Ronquist 2001) plugin of GENEIOUS®PRO v5.6.6, we used a model with the closest likelihood available (GTR + I + Γ). Two parallel runs were performed in MRBAYES using four chains (one cold and three hot) for 1.1 × 10^6^ generations and sampling every 200 generations from the Markov Chain Monte Carlo (MCMC). We determined stationarity by plotting the log likelihood scores of sample points against generation time; when the values reached a stable equilibrium and split frequencies fall below 0.01, stationarity was assumed. We discarded 100,000 samples and 10% of the trees as burn-in. A maximum clade credibility (MCC) tree was constructed using TREEANNOTATOR v1.7.5 (Drummond et al. 2012). We consider Bayesian Posterior Probabilities (BPP) >95% as evidence of significant support for a clade (Huelsenbeck and Ronquist 2001, Wilcox et al. 2002).

### Morphological data and analyses

A total of 199 individuals (see species descriptions and Data resources below) representing three putative different populations and four Peruvian species (*Liolaemus alticolor*, *Liolaemus incaicus*, *Liolaemus tacnae* and *Liolaemus walkeri*) assigned to the *Liolaemus alticolor* group were scored for three classes of morphological characters. We performed a character analysis of 17 discrete binomial characters related to scalation, pattern of coloration and skin folds, including the following: presence/absence of smooth (1) temporal scales and (2) dorsal head scales, contact or not of (3) rostral to nasal scale, presence/absence of (4) mucronate dorsal scales and (5) precloacal pores, (6) preocular scale same or different color as loreal region, presence/absence of (7) spots on dorsal head scales, (8) black line surrounding the interparietal scale, regular spots or marks in (9) paravertebral field and (10) lateral field, presence/absence of dorsolateral stripes (11) and vertebral line (12), marks or spots on throat (13), melanistic belly (14), ringed pattern in ventral tail (15), and presence/absence of antehumeral (16) and neck folds (17). All characters were scored using a stereomicroscope and from photos of live animals taken in the field.

For statistical analyses of these discrete variables we used tolerance intervals as described in the tolerance package of Young (2010), which in a random sample of a univariate population, is an interval expected to contain a specified proportion or more of the sampled population (Krishnamoorthy and Mathew 2009). We used binomial tolerance intervals to estimate the number of individuals that comprise 95% of the population expected to have one state with a 0.05 level of significance (following Wiens and Servedio 2000, Zapata and Jiménez 2012). One-sided binomial tolerance intervals were estimated using the Wilson method (WS), which is appropriate when the sample sizes are small (n ≤ 40) (Young 2010).

We scored the following 11 morphometric characters: (SVL) snout-vent length, (AGL) axilla-groin length (between the posterior insertion of forelimb and anterior insertion of thigh), (HL) head length (from snout to anterior border of auditory meatus), (HW), head width (at widest point), (FOL) forelimb length (distance from the attachment of the limb to the body to the terminus of the fourth digit), (HIL) hindlimb length (distance from the attachment of the limb to the body, to the terminus of the fourth digit), (SL) snout length (from snout to anterior border of eye), (AMW) auditory meatus width, (AMH) auditory meatus height, (RW) rostral width, and (RL) rostral length. We also scored five meristic characters, including: (MBS) number of midbody scales (counted transversely at the middle of the body), (DTS) dorsal trunk scales (counted from the level of anterior border of ear to anterior border of thighs), (DHS) dorsal head scales (counted from the rostral scale to anterior border of ear), (VS) ventral scales (counted from the mental scales to the cloaca), and (SCI) number of scales in contact with the interparietal. Measurements and counts were taken from the right side of the animal using a stereomicroscope. Morphometric data were only taken for adult males and females.

After testing for normality in all morphometric and meristic characters with the Shapiro-Wilks test (Shapiro and Francia 1972), we summarized means and ranges for all population samples, and performed Principal Component Analyses (PCA) and Correspondence Analyses (CA) separately for each class of characters and by sex, to summarize patterns. Results of PCA and CA were then compared with the analysis of continuous characters by estimating normal tolerance intervals to find gaps or discontinuities in each class of morphological characters. We used normal tolerance intervals to estimate the lowest and highest values of a continuous character that is contained in 95% of the population with a 0.05 level of significance. Two-sided normal tolerance intervals were estimated using the Howe method (HE), which is considered to be extremely accurate, even for small sample sizes (Young 2010).

For comparison with normal tolerance intervals we assessed the morphometric and meristic characters with univariate ANOVA and Mann-Whitney *U* tests for parametric and non-parametric distributions, respectively. When the assumption of equal-variance was not met for an ANOVA test, the unequal-variance (Welch) version of ANOVA was performed. Each character was tested for intersexual differences, and if present, the sexes were analyzed separately. Results were considered significant when p ≤ 0.05. However, we didn’t use the results of the ANOVA and Mann-Whitney *U* tests in our taxonomic decisions (see Introduction and Discussion). Binomial and tolerance intervals were calculated with the package Tolerance (Young 2010) in R v3.0.1 (R core team 2013). Test of normality, PCA, CA and univariate tests were performed using PASTv. 2.08b, (Hammer et al. 2001).

### Distributional models

We used the maximum entropy model implemented in the program MAXENT v3.3.3e (Phillips et al. 2006) to predict where the Peruvian lineages of *Liolaemus walkeri* complex are most likely to occur under current climatic conditions. MAXENT generates distributional models (or ecological niche models; ENMs) using presence-only records, contrasting them with background/pseudoabsence data sampled from the remainder of the study area. We chose this approach because of its overall better performance with presence-only data and with small sample sizes (Elith et al. 2006). ENMs were developed from occurrence points used in this study, and records without duplicates are: 22 for Ancash, 31 for Ayacucho, 16 for Cusco, 33 for *Liolaemus tacnae* and 52 for *Liolaemus walkeri* (see Data resources below). For niche predictions, we used the 19-bioclimatic variables from the WorldClim v1.4 dataset with a resolution of 2.5 min (Hijmans et al. 2005). Bioclimatic variables were derived from monthly temperature and precipitation layers, and represents biologically meaningful properties of climate variation (Hijmans et al. 2005). Layers were trimmed to the areas surrounding each species and populations that might represent new species, and then projected over a larger region (-9.828° to -17.839° and -77.486° to -69.811°).

For model calibration we used the default settings with 1000 iterations, and the minimum training value averaged over the 10 replicates as threshold with the default convergence threshold (10^–5^). Due to our smaller samples sizes, we used for model calibration and performance the cross-validation option with 10 replicates, and average the results to estimate species niche and distributions. For model significance, 25% of localities were randomly set aside as test points and the area under the curve (AUC) was calculated, which summarizes the model’s ability to rank presence localities higher than a sample of random pixels (Peterson et al. 2011). AUC values ≤ 0.5 correspond to predictions that are equal or worse than random. AUC values > 0.5 are generally classed into (1) poor predictions (0.5 to 0.7); (2) reasonable predictions (0.7 to 0.9); and (3) very good predictions (>0.90; but see Peterson et al. 2011, for caveats on use of AUC in presence/background data). Model clamping was checked with the “fade by clamping” option available in MAXENT v 3.3.3e. Estimates of bioclimatic variable importance was performed using the Jackknife test. We used the logistic output (probability values) and mapped the distributional models showing areas from the average minimum logistic values (threshold) to 1 as areas suitable for species.

Schoener’s D metric was introduced as a measure of niche similarity between pairs of populations (or species) by Warren et al. (2010), and is calculated using the ENMTOOLS package. We calculated these values by comparing the climatic suitability of each grid cell in the projected area obtained with MAXENT. This similarity measure ranges from 0 (niche models have no overlap) to 1 (niche models identical; Warren et al. 2008). We estimated similarity measures and then tested whether the ENMs produced by two populations or species are identical using the niche identity test in ENMTOOLS. One hundred pseudoreplicate data sets were generated to obtain a distribution of D scores, and we reject the hypothesis of niche identity when the empirically observed value for D is significantly lower than the values expected from the pseudoreplicated data set (Warren et al. 2010).

### Species descriptions

Species descriptions follow the terminology of Lobo and Espinoza (1999) and Quinteros (2013). For diagnosis, we selected the following non-Peruvian species assigned to the *Liolaemus alticolor* group: *Liolaemus aparicioi* Ocampo, Aguilar-Kirigin & Quinteros, *Liolaemus bitaeniatus* Laurent, *Liolaemus chaltin* Lobo & Espinoza, *Liolaemus pagaburoi* Lobo & Espinoza, *Liolaemus paulinae* Donoso-Barros, *Liolaemus puna* Lobo & Espinoza, *Liolaemus pyriphlogos* Quinteros, and *Liolaemus variegatus* Laurent. This selection is based on previous phylogenetic analyses (Espinoza et al. 2004, Díaz-Gómez and Lobo 2006, Schulte and Moreno-Roark 2010, Quinteros 2013), and taxonomic revisions and species descriptions of geographically proximate species (Donoso-Barros 1961, Laurent 1984, Lobo and Espinoza 1999, 2004, Quinteros 2012, Ocampo et al. 2012). We assumed that diagnostic characters are “fixed”. Color descriptions are based on photographs of live animals taken in the field, and specimens examined are provided in Data resources.

## Data resources

The data underpinning the analysis reported in this paper are deposited in the Dryad Data Repository at http://doi.org/10.5061/dryad.0q7pc, and at GBIF, the Global Biodiversity Information Facility, http://ipt.pensoft.net/ipt/resource.do?r=ocurrence_records_liolaemus_walkeri_complex.

## Results

### Phylogenetic Analysis

**Figure 1.**
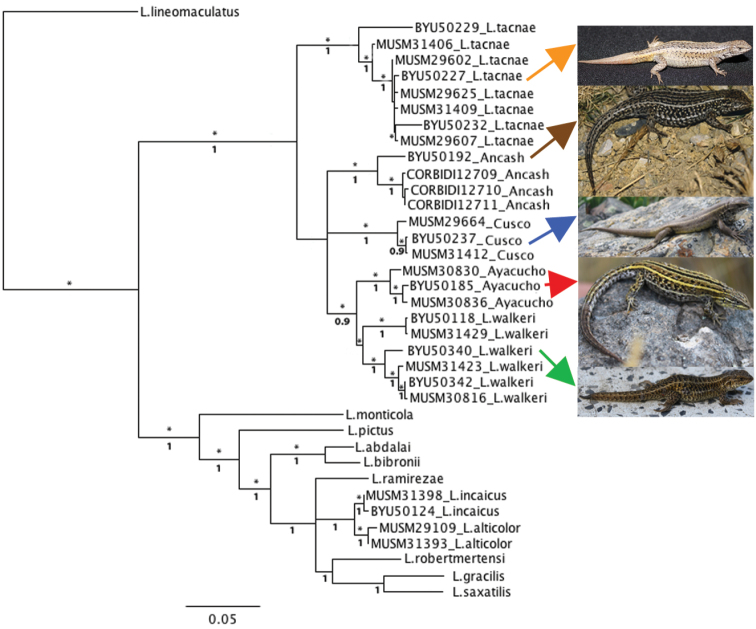
Concatenated maximum likelihood (-Log L = 8452.31415) tree based on cyt-b and 12S haplotypes of focal taxa (Ancash, Ayacucho Cusco) and species assigned to the *alticolor* group and outgroups. Bootstrap ≥ 70 (*) and posterior probabilities values are shown above and below branches respectively.

A tree with maximum likelihood bootstrap values (logL = -8452.31415, MLB) and Bayesian posterior probabilities (BPP) based on 1421 aligned base pairs is shown in Fig. 1. Differences between both methods are mentioned below. Both ML and Bayesian analyses recovered Ancash, Ayacucho, Cusco, *Liolaemus tacnae* and *Liolaemus walkeri* haplotypes as monophyletic groups with high support. Both also showed a close relationship between Ayacucho and *Liolaemus walkeri* haplotypes, but relationships between Ancash, Cusco and the (*Liolaemus walkeri* + Ayacucho) clade were unresolved and with moderate support in the ML tree (MLB 65%). The Bayesian analysis recovers Ancash as the sister to the (*Liolaemus walkeri* + Ayacucho) clade with low support (BPP 0.5), and Cusco as the sister clade to the ((*Liolaemus walkeri* + Ayacucho) Ancash) clade with moderate support (BPP 0.9). In both analyses, *Liolaemus tacnae* is recovered as the sister group of the (Ancash + Cusco + (*Liolaemus walkeri* + Ayacucho)) clade with moderate support (MLB 65%, BPP 0.9). *Liolaemus tacnae* and *Liolaemus walkeri* are assigned to the *alticolor-bibronii* group, but the clade (*Liolaemus tacnae* (Ancash + Cusco + (*Liolaemus walkeri* + Ayacucho))) is strongly differentiated from the other species assigned to the *alticolor-bibronii* group (Fig. 1).

The monophyletic group (*Liolaemus tacnae* (Ancash + Cusco + (*Liolaemus walkeri* + Ayacucho))) is the sister group of a clade comprised of taxa assigned to different species groups in the subgenus *Liolaemus*, including species of the *alticolor-bibronii* group. The relationships of these two more inclusive clades showed high MLB, but low BPP values. In this clade, both ML and Bayesian analyses recovered *Liolaemus alticolor* and *Liolaemus incaicus* haplotypes as monophyletic groups with high support. In our ML analysis, the clade (*Liolaemus alticolor* + *Liolaemus incaicus*) has unresolved relationships with *Liolaemus ramirezae* and the clade (*Liolaemus robertmertensi* + (*Liolaemus gracilis* + *Liolaemus saxatilis*)), and this latter clade has high BPP but low MLB support (Fig. 1). *Liolaemus abdalai* and *Liolaemus bibronii* are recovered as sister taxa with high support, and this clade is sister to the clade (*Liolaemus ramirezae* + (*Liolaemus incaicus* + *Liolaemus alticolor*) + (*Liolaemus robertmertensi* + (*Liolaemus gracilis* + *Liolaemus saxatilis*))) also with high support (Fig. 1). *Liolaemus pictus* is sister to the clade ((*Liolaemus abdalai* and *Liolaemus bibronii*) + (*Liolaemus ramirezae* + (*Liolaemus incaicus* + *Liolaemus alticolor*) + (*Liolaemus robertmertensi* + (*Liolaemus gracilis* + *Liolaemus saxatilis*)))), and *Liolaemus monticola* is basal to a clade that includes *Liolaemus pictus* and its sister group.

### Morphological analyses
Binomial discrete characters

Because our phylogenetic analysis did not show a close relationship between (*Liolaemus alticolor* + *Liolaemus incaicus*)and the (*Liolaemus tacnae* (Ancash + Cusco + (*Liolaemus walkeri* + Ayacucho))) clades, we focus our comparisons on these last five taxa. Of the 17 binomial characters, four were useful for species delimitation among these taxa (Table 1). One-sided binomial tolerance intervals (BTI) for 95% of the population with a 0.05 level of significance is indicated below for each of these four characters.

**Figure 2.**
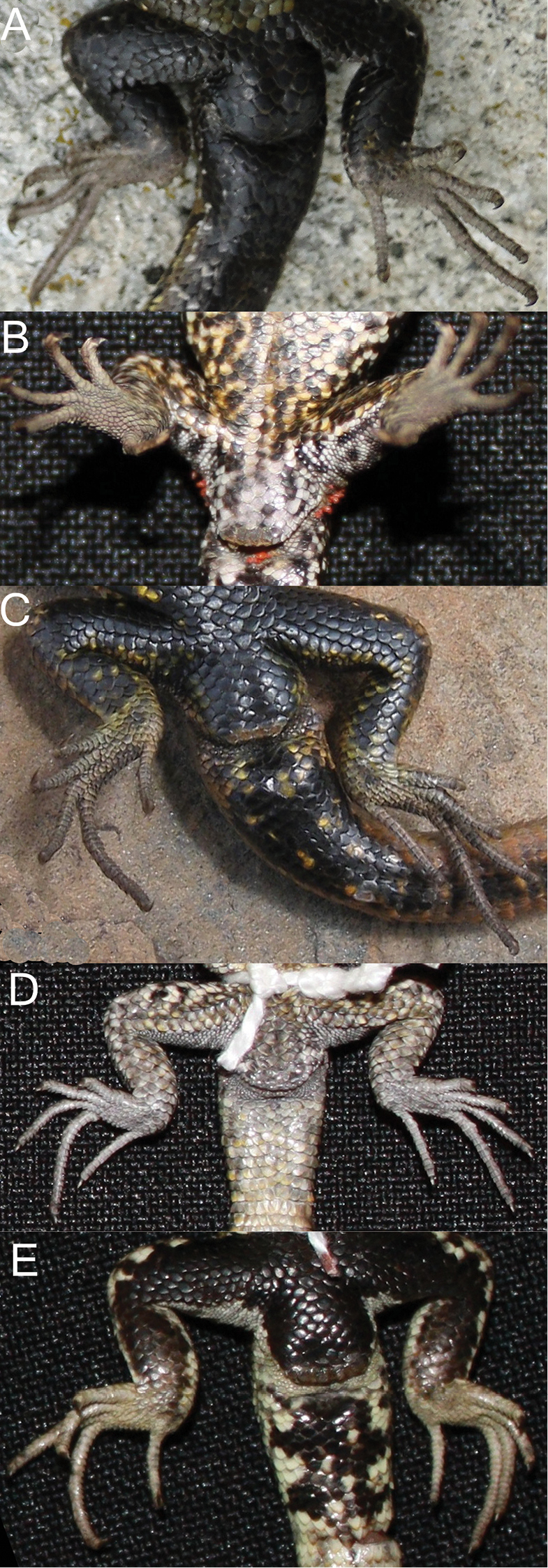
Detailed view of the cloaca region showing absence (**A, D**) or presence (**B, C, E**) of precloacal pores: **A** Ancash **B** Ayacucho **C** Cusco **D** *Liolaemus tacnae* and **E** *Liolaemus walkeri*.

Ancash (n = 12) and *Liolaemus tacnae* (n = 18) males differed from Ayacucho, Cusco and *Liolaemus walkeri* males in lacking precloacal pores (Fig. 2A and D; vs. presence in panels B, C, and E). Although these differences are fixed in our samples, the BTI tests showed that up to 36% and 31% of the Ancash and *Liolaemus tacnae* populations, respectively, have a significant probability of possessing the alternative state (P ≤ 0.05) in a larger sample.

**Table 1.** Binomial characters for females (F) and males (M) of focal populations of *Liolaemus* lizards sampled for this study. Character states useful for species discrimination are in bold, and states only assessed on adults are indicated with an asterisk.

	Ancash		Ayacucho		Cusco		*Liolaemus tacnae*		*Liolaemus walkeri*	
	F (n=18)	M (n=12)	F (n=18)	M (n=10)	F (n=8)	M (n=8)	F (n=23)	M (n=18)	F (n=48)	M (n=21)
Temporal scales smooth	yes	yes/no	yes/no	yes/no	yes	yes	yes/no	yes	yes	yes/no
Dorsal surface of head completely smooth	yes/no	yes/no	yes/no	yes/no	yes	yes/no	yes/no	yes	yes/no	yes/no
Nasal contact rostral scale	yes/no	yes/no	yes/no	yes	yes/no	yes/no	yes/no	yes/no	yes	yes/no
Dorsal scales mucronate	no	no	yes/no	yes/no	no	no	no	no	no	no
**Precloacal pores**	no	**no**	no	**yes**	no	**yes**	no	**no**	no	**yes**
Sub and preoculars different in color from loreal region	yes/no	yes/no	yes/no	yes/no	yes/no	yes/no	yes/no	yes/no	yes/no	yes/no
Dorsal surface of head with marks or dots	yes	yes	yes/no	yes/no	yes/no	yes	yes/no	yes/no	yes/no	yes/no
Black line surrounds interparietal scale	yes/no	yes/no	yes/no	yes/no	yes/no	yes/no	yes/no	yes/no	yes/no	yes/no
Regular marks or spots in paravertebral field	yes/no	no	yes	yes/no	yes/no	no	yes/no	yes/no	yes/no	yes/no
**Regular marks or spots in lateral field**	**yes**	**yes**	**yes**	**yes**	**no**	**no**	yes/no	yes/no	yes/no	yes/no
Dorsolateral stripes	yes	yes/no	yes	yes	yes	yes	yes	yes/no	yes	yes
Vertebral line	yes	yes	yes	yes	yes	yes	yes/no	yes/no	yes	yes/no
Throat not immaculate	yes/no	yes/no	yes/no	yes/no	yes	yes	yes/no	yes	no	yes
*Complete or partial melanistic belly	yes/no	**yes**	no	yes/no	no	**yes**	no	no	no	**yes**
*Ventral tail with ringed pattern	yes/no	yes/no	yes/no	**yes**	no	**no**	no/yes	yes	yes/no	**no**
Antehumeral fold	yes	yes	yes	yes	yes	yes	yes	yes	yes	yes
Neck folds	yes	yes	yes	yes	yes	yes	yes	yes	yes	yes

**Figure 3.**
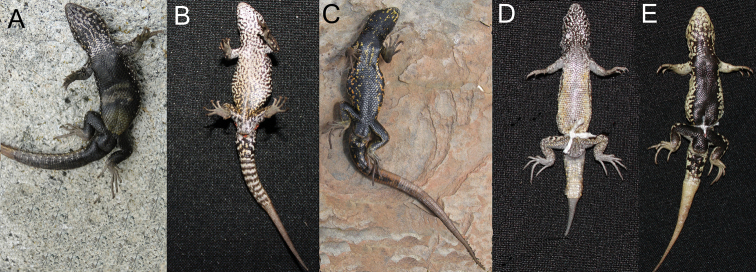
Ventral view showing the color patterns of the belly and tail: **A** Ancash **B** Ayacucho **C** Cusco **D** *Liolaemus tacnae* and **E** *Liolaemus walkeri*.

Adult males of Ancash (Fig. 3A) differ from *Liolaemus tacnae* (Fig. 3D) in having a melanistic belly, and again while fixed in our samples, BTI showed that up to 36% of the population may have the alternative state (P ≤ 0.05). Adult males of Ayacucho (Fig. 3B) can be diagnosed by their ringed ventral tail pattern, in contrast to the other four samples (Fig. 3A, C–E), but up to 44% of the population may have the alternative state (P ≤ 0.05).

**Figure 4.**
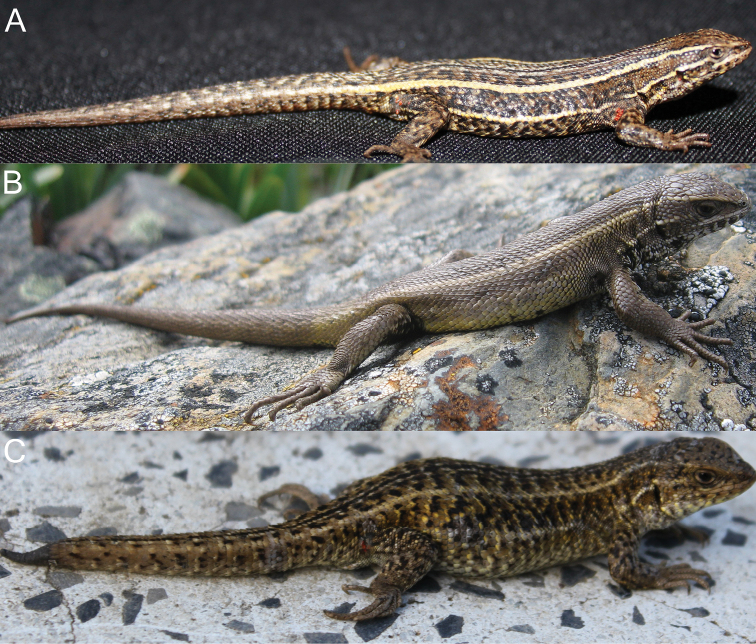
Lateral view showing the color patterns of **A** Ayacucho **B** Cusco, and **C** *Liolaemus walkeri*.

Both sexes of the Cusco sample (n = 16; Fig. 4B) differed from all Ayacucho (n = 28; Fig. 4A) and most individuals (90% of n = 69; Fig. 4C) of *Liolaemus walkeri*, in lacking regular spots or marks in lateral fields; but up to 33% of the population may have the alternative state (P ≤ 0.05).

### Morphometric and meristic characters

Our empirical results are summarized in Table 2, and tolerance intervals are given in Tables 3 and 4 for morphometric and meristic variables, respectively. Statistical tests rejected normality for HW, AMW, RW and all meristic characters, but we assumed normality because our sample sizes were too small to implement non-parametric tolerance interval tests. We did not find any diagnostic character or gaps in either data set (Tables 3 and 4).

**Table 2.** Descriptive statistics of morphometric and meristic characters for three new species of *Liolaemus* described herein, and *Liolaemus tacnae* and *Liolaemus walkeri*. First rows show ranges and second rows show means and standard deviations. See methods for abbreviations.

	*Liolaemus chavin* (Ancash, n=32)	*Liolaemus pachacutec* (Cusco, n=18)	*Liolaemus tacnae* (n=41)	*Liolaemus walkeri* (n=78)	*Liolaemus wari* (Ayacucho, n=30)
SVL	51.0–66.5	33.4–52.0	42.6–56.6	41.5–64.4	50.0–61.4
	57.0±4.0	45.4±4.4	48.6±3.2	54.5±4.6	55.6±3.1
AGL	20.4–34.8	17.8–30.8	14.9–26.5	17.2–33.5	19.8–32.3
	26.5±3.4	22.6±3.6	21.8±2.7	25.3±3.4	25.9±3.9
HL	10.2–15.3	9.2–13.2	9.4–12.0	10.1–14.2	10.3–12.7
	12.4±1.1	10.6±1.0	10.7±0.7	12.2 ±0.9	11.4±0.8
HW	8.8–12.8	6.6–9.7	7.2–9.3	7.8–11.6	8.1–10.7
	10.3±1.1	8.2±0.7	9.6±0.8	9.6±0.9	9.4±0.8
SL	4.2–6.3	3.2–4.7	3.7–5.3	3.5–6.9	4.0–4.9
	5.2±0.5	4.0±0.5	4.5±0.4	5.1±0.5	4.4±0.3
FoL	14.1–19.1	12.9–17.4	13.1–8.3	13.5–21.5	13.9–18.3
	16.2±1.5	15.7±1.3	15.7±1.4	16.7±1.5	15.7±1.2
HiL	22.6–29.5	19.0–27.9	20.8–29.8	19.8–30.7	20.9–28.7
	25.8±1.8	23.4±2.1	24.6±2.2	25.2±2.5	24.2±2.5
AMH	1.7–2.9	1.3–2.4	1.5–2.5	1.4–2.6	1.7–2.5
	2.2±0.26	1.8±0.3	1.9±0.2	2.1±0.3	2.1±0.22
AMW	0.70–1.31	0.8–1.3	0.5–1.5	0.6–1.6	0.76–1.30
	1.0±0.2	1.0±0.1	1.2±0.1	1.2±0.2	1.1±0.1
RH	0.8–1.3	0.6–2.4	0.8–1.3	0.7–1.6	0.9–1.2
	1.0±0.1	1.0±0.3	1.0±0.1	1.1±0.2	1.0±0.1
RW	2.2–3.2	2.1–2.7	1.6 –2.8	1.9–3.1	2.0–2.9
	2.7±0.3	2.6±0.1	2.2±0.2	2.6±0.3	2.5±0.3
MBS	48–69	39–51	42–58	45–60	46–56
	56.8±6.1	46.5.6±3.4	48.1±4.1	53.8±3.6	50.6±3.0
DTS	43–72	42–57	40–55	42–66	40–55
	56.1±7.2	47.2±3.6	47.0±4.1	54.4±4.6	46.4±3.6
DHS	10–19	10–16	11–18	10–19	9–17
	14.6±2.1	13.5±1.5	14.0±1.7	13.7±1.7	12.7±1.8
VS	70–87	56–82	60–87	69–96	71–88
	79.6±4.5	72.8±6.4	76.3±6.5	80.7±5.2	77.7±4.1
SCI	5–12	4–8	5–10	5–9	5–13
	7.9±1.4	6.4±1.2	7.0±1.0	7.1±1.0	7.6±1.4

**Table 3.** Normal tolerance intervals for morphometric variables of three species of *Liolaemus* described herein, plus *Liolaemus tacnae* and *Liolaemus walkeri*; those identified with an asterisk were assumed to follow a normal distribution. See methods for abbreviations.

	Ancash (n=29)	Ayacucho (n=16)	Cusco (n=17)	*Liolaemus tacnae* (n=36)	*Liolaemus walkeri* (n=74)
SVL	46.7–67.3	46.5–64.6	32.7–58.1	40.5–56.8	44.0–65.1
AGD	17.7–35.3	14.4–37.3	12.3–32.9	15.1–28.6	17.5–33.0
HL	9.6–15.2	9.1–13.8	7.7–13.5	9.1–12.3	10.1–14.3
*HW	7.4–13.1	7.1–11.6	6.0–10.3	7.0–10.0	7.5–11.8
SL	4.0–6.4	3.6–5.2	2.6–5.3	3.5–5.6	3.9–6.4
FoL	12.4–19.9	12.2–19.2	11.9–19.5	12.8–19.0	13.4–20.3
HiL	21.2–30.4	17.1–31.4	17.5–29.3	19.2–30.0	19.5–31.0
AMH	1.6–2.9	1.5–2.7	0.9–2.6	1.4–2.4	1.4–2.8
*AMW	0.6–1.4	0.6–1.5	0.6–1.4	0.6–1.7	0.8–1.7
RH	0.7–1.4	0.8–1.3	0.2–2.1	0.7–1.3	0.7–1.6
*RW	1.9–3.5	1.8–3.3	1.8–2.9	1.6–2.8	2.0–3.2

**Table 4.** Normal tolerance intervals for meristic characters of three species of *Liolaemus* described herein, plus *Liolaemus tacnae* and *Liolaemus walkeri*; all variables were assumed to follow a normal distribution. See methods for abbreviations.

	Ancash (n=32)	Ayacucho (n=30)	Cusco (n=18)	*Liolaemus tacnae* (n=42)	*Liolaemus walkeri* (n=79)
MBS	41.4–72.3	43.1–58.2	36.9–56.1	38.0–58.2	45.6–62.0
DTS	38.0–74.2	37.2–55.7	36.9–57.5	37.0–57.0	43.8–65.3
DHS	9.2–20.0	8.3–17.4	9.2–17.9	9.8–18.3	9.9–17.5
VS	68.2–91.0	67.1–88.3	54.7–91.0	60.5–92.2	68.9–92.5
SCI	4.5–11.3	4.1–11.2	3.0–9.8	4.5–9.5	4.8–9.4

**Figure 5.**
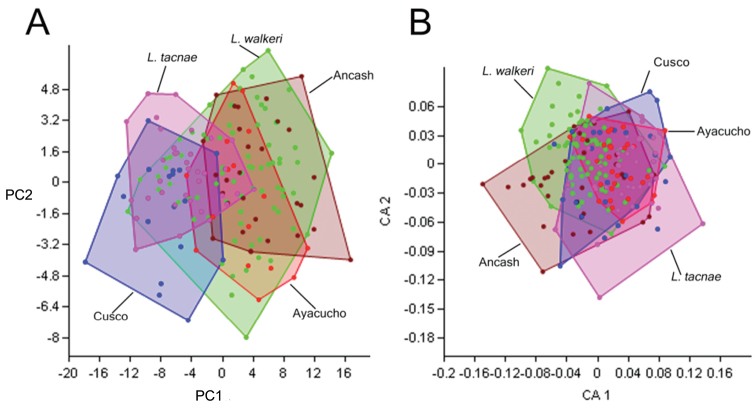
First and second principal components (PC) and correspondence axes (CA) of morphometric (**A**) and meristic (**B**) data of Ancash, Ayacucho, Cusco, *Liolaemus tacnae* and *Liolaemus walkeri* respectively.

Principal Component and Correspondence Analyses separated by sex or pooled together did not show any differences, so we present the results of the pooled analyses. Principal Component (PC) Analysis revealed that PC1 and PC2 explained 90% of the variance, and the Correspondence Analysis revealed that Correspondence Axis (CA) 1 and CA2 explained 66% of the similarity for morphometric and meristic data, respectively (see also Supplementary file 4 for corresponding eigenvalues, and percentages of variance and similarity accounted by principal components and correspondence axes). The bivariate plot for the morphometric variables revealed extensive overlap of *Liolaemus walkeri* with the remaining four samples, but minimal overlap between the Ancash and Cusco samples, and little overlap between the Ayacucho and Cusco (Fig. 5A). Both of these pairs are differentiated primarily along PC1, for which SVL and AGL contributed the highest loadings (0.85 and 0.47 respectively). The Cusco samples are characterized by shorter SVL and axilla-groin lengths than the Ancash and Ayacucho samples. The PC analyses revealed extensive overlap among all samples along PC2, and the CA for the meristic variables (Fig. 5B) revealed extensive overlap among all five samples along both axes.

Only significant results of ANOVA are mentioned below and the sex of a particular species or population is indicated only if significantly different from the opposite sex. For SVL, there were significant differences between Ancash vs. Cusco, *Liolaemus tacnae* and *Liolaemus walkeri*; Ayacucho vs. Cusco and *Liolaemus tacnae*; Cusco vs. *Liolaemus tacnae* and *Liolaemus walkeri*.

For AGD, there were significant differences between Ancash males vs. Cusco males and *Liolaemus tacnae* males; Ancash females vs. *Liolaemus tacnae* females; Ayacucho females vs. Cusco females and *Liolaemus tacnae* females; Cusco males vs. *Liolaemus tacnae* males and *Liolaemus walkeri* males; Cusco females vs. *Liolaemus walkeri* females.

For HL, there were significant differences between Ancash males vs. Ayacucho males, Cusco, *Liolaemus tacnae* and *Liolaemus walkeri* males; Ancash females vs. Ayacucho females, Cusco, *Liolaemus tacnae* and *Liolaemus walkeri* females; Ayacucho males vs. Cusco and *Liolaemus tacnae*; Ayacucho femalesvs. *Liolaemus walkeri* females; Cuscovs. *Liolaemus walkeri* males and *Liolaemus walkeri* females.

For FoL, there were significant differences between Ancash males vs. Cusco and *Liolaemus tacnae*; Ancash females vs. *Liolaemus walkeri*; Ayacucho females vs. *Liolaemus walkeri*; Cuscovs. *Liolaemus walkeri*.

For HiL, there were significant differences between Ancash males vs. Ayacucho, Cusco males and *Liolaemus tacnae*; Ancash females vs. Cusco females; Ayacuchovs. Cusco females and *Liolaemus walkeri* males; Cusco females vs. *Liolaemus tacnae* and *Liolaemus walkeri* females.

For SL, there were significant differences between Ancash males vs. Ayacucho males, Cusco, *Liolaemus tacnae* and *Liolaemus walkeri*; Ancash females vs. Ayacucho females and Cusco; Ayacucho males vs. Cusco; Ayacucho malesand females vs. *Liolaemus walkeri*; Cusco vs. *Liolaemus tacnae* and *Liolaemus walkeri*;

For AMH, there were significant differences between Ancash vs. Cusco, *Liolaemus tacnae*, *Liolaemus walkeri*; Ancash vs. Ayacucho females; Ayacucho males vs. Cusco and *Liolaemus tacnae*; Ayacucho females vs. Cusco; Cusco vs. *Liolaemus tacnae* and *Liolaemus walkeri*.

For RH, there were significant differences between Ancash males vs. *Liolaemus tacnae*; Ancash females vs. *Liolaemus walkeri*; Ayacucho females vs. *Liolaemus walkeri*; Cusco vs. *Liolaemus walkeri*.

Only significant results of Mann-Whitney *U* are mentioned below and the sex of a particular species or population is indicated only if significantly different from the opposite sex. For HW, there were significant differences between Ancash males vs. Ayacucho, Cusco, *Liolaemus tacnae* and *Liolaemus walkeri*; Ancash females vs. Cusco and *Liolaemus tacnae*; Ayacucho vs. Cusco and *Liolaemus tacnae*; Cusco vs. *Liolaemus walkeri*.

For AMW, there were significant differences between Ancash vs. Cusco; Ayacucho vs. *Liolaemus tacnae*; Cusco vs. *Liolaemus tacnae* and *Liolaemus walkeri*.

For RW, there were significant differences between Ancash males vs. Cusco, *Liolaemus tacnae* males, and *Liolaemus walkeri*; Ancash females vs. Cuscoand *Liolaemus tacnae* females; Ayacucho males vs. Cusco and *Liolaemus tacnae* males; Ayacucho females vs. *Liolaemus tacnae* females and *Liolaemus walkeri*; Cusco vs. *Liolaemus tacnae* females and *Liolaemus walkeri*.

For MBS, there were significant differences between Ancash vs. Ayacucho, Cusco, *Liolaemus tacnae* and *Liolaemus walkeri*; Ayacucho vs. Cusco, *Liolaemus tacnae* and *Liolaemus walkeri*; Cuscovs. *Liolaemus walkeri*.

For DTS, there were significant differences between Ancashvs. Ayacucho, Cusco, *Liolaemus tacnae*, *Liolaemus walkeri* males and *Liolaemus walkeri* females; Ayacucho vs. *Liolaemus walkeri* males and *Liolaemus walkeri* females; Cusco vs. *Liolaemus walkeri* males and *Liolaemus walkeri* females.

For DHS, there were significant differences between Ancash vs. Ayacucho females, Cusco females and *Liolaemus walkeri*; Ayacucho females vs. *Liolaemus tacnae* and *Liolaemus walkeri*; Cusco females vs. *Liolaemus tacnae*.

For VS, there were significant differences between Ancash vs. Cusco and *Liolaemus tacnae*; Ayacucho vs. Cusco and *Liolaemus walkeri* females; Cusco vs. *Liolaemus tacnae*, *Liolaemus walkeri* males and *Liolaemus walkeri* females.

For SCI, there were significant differences between Ancash males vs. Ayacucho, Cusco, *Liolaemus tacnae* and *Liolaemus walkeri*; Ancash females vs. Cusco; Ayacucho vs. Cusco and *Liolaemus tacnae*; Cusco vs. *Liolaemus walkeri*.

### Distributional models

**Figure 6.**
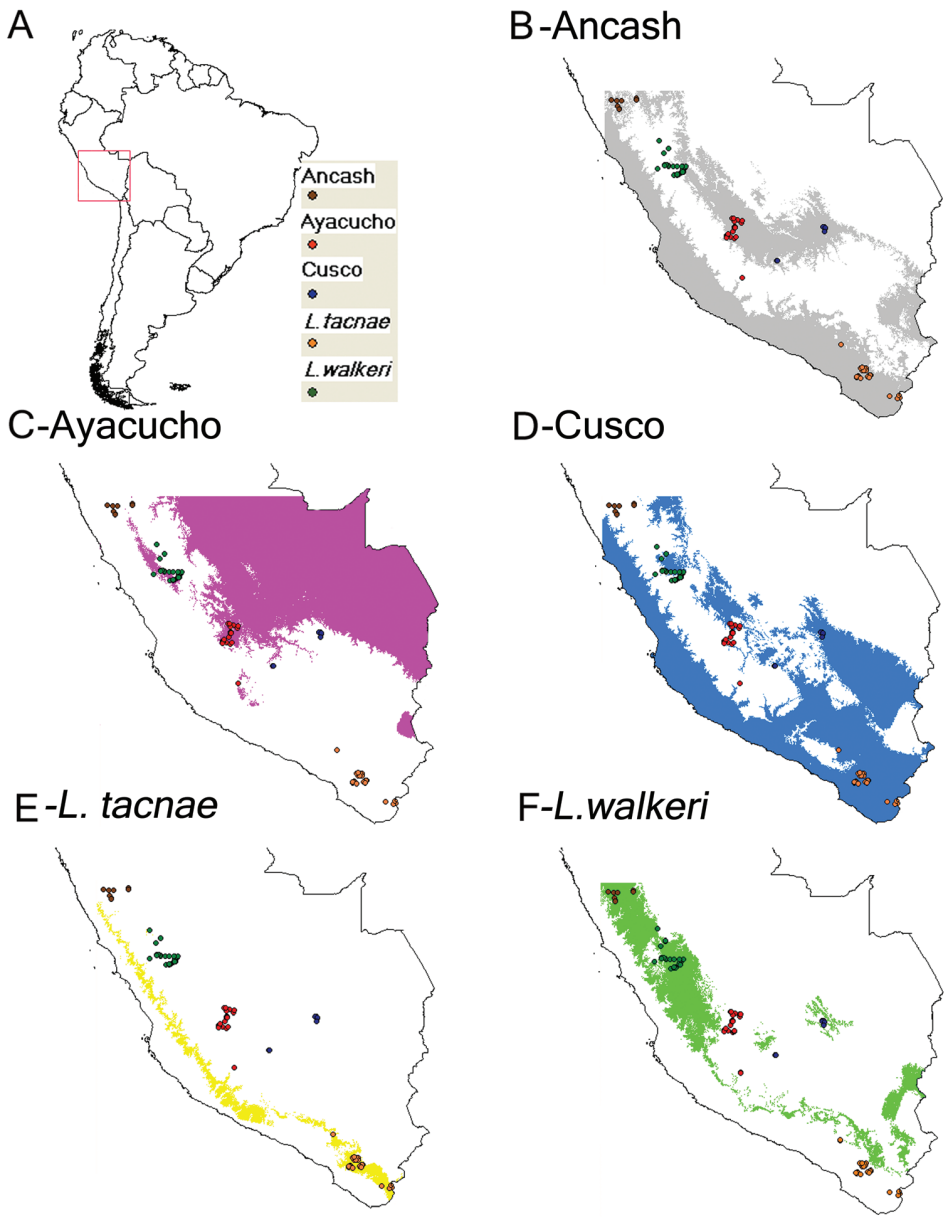
Predicted area and known geographic distribution (**A**) used to develop distributional models of Ancash (**B**) Ayacucho (**C**) Cusco (**D**) *Liolaemus tacnae* (**E**) and *Liolaemus walkeri* (**F**).

The predicted distribution in all cases matched the known range of each taxon, although some of these overlap. However, the distributional models of Ayacucho vs *Liolaemus tacnae* (Fig. 6; C vs. E), as well as those for *Liolaemus walkeri* and *Liolaemus tacnae* (Fig. 6; E vs. F) are virtually mutually exclusive. All other combinations of distributional models overlapped, but differed in the contribution of bioclimatic variables to each niche envelope, and in predicting the known distribution of particular taxa (Table 5, Fig. 6). For example, the most important bioclimatic variables for the Ancash model were completely different from those for the *Liolaemus walkeri* and Ayacucho models (Table 5). In the same manner, the most important bioclimatic variables contributing to the Ayacucho model were completely different from those for the *Liolaemus walkeri* and Cusco models (Table 5). The most important bioclimatic variables for the Cusco model were completely different to those for *Liolaemus tacnae* (Table 5). Moreover results from the Niche Identity Test found all pairwise comparison between focal populations and species significantly different, except for Ancash and Cusco (Table 6).

**Figure 7.**
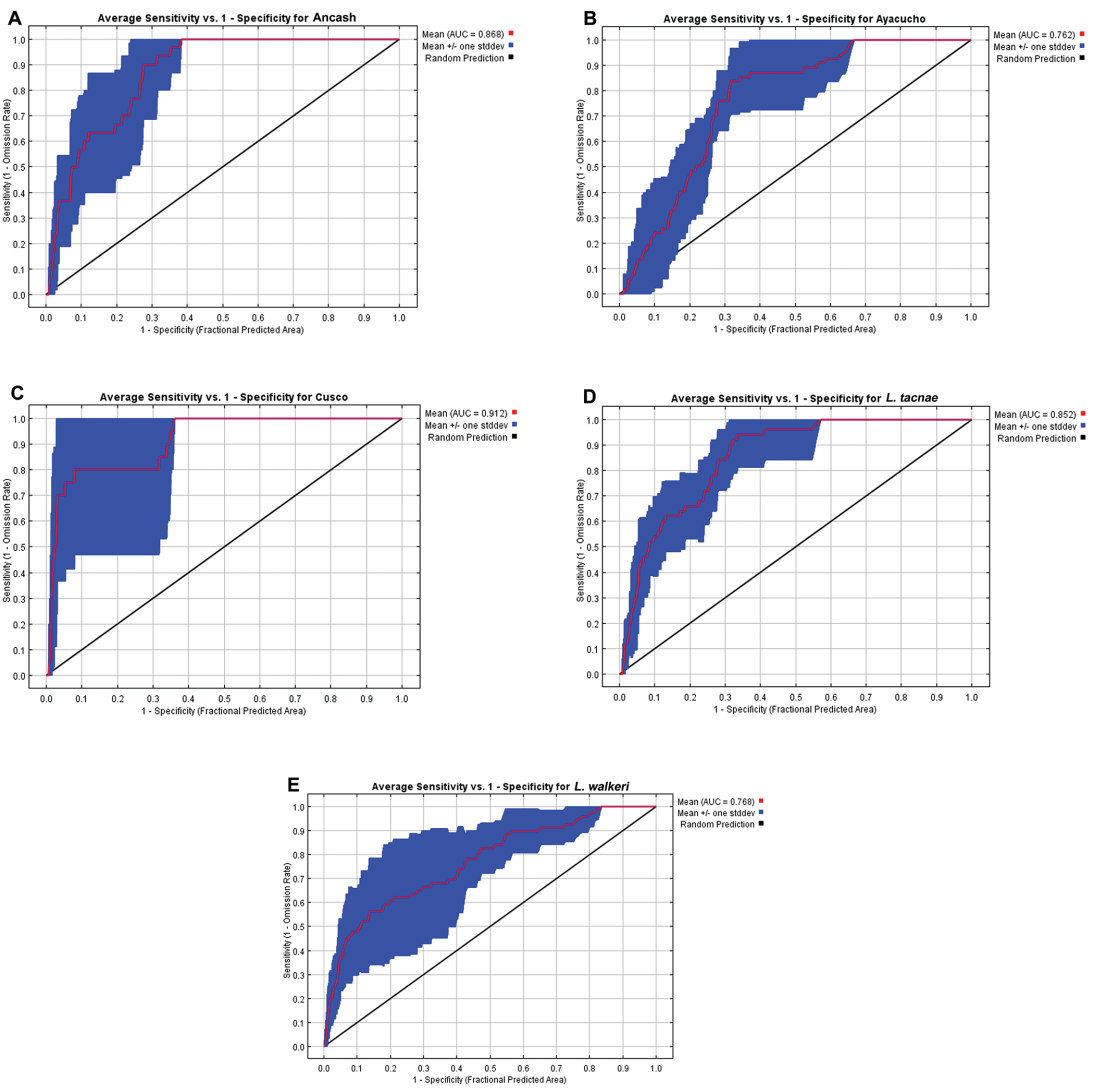
Receiver operating characteristic curves and AUC values for **A** Ancash **B** Ayacucho **C** Cusco **D** *Liolaemus tacnae* and **E** *Liolaemus walkeri*.

The Ancash model (Fig. 6B) overlapped the known geographic distributions of Ayacucho, Cusco, *Liolaemus tacnae*, and partially with *Liolaemus walkeri*, but the two most important bioclimatic variables accounting for 94.3% of the contribution to this model were Precipitation of Warmest Quarter (63.3%) and Isothermality (31.0%; Table 5). These were also the most important variables in the permutation and jackknife tests. Thus the Ancash samples are characterized by a niche envelope with relative lower precipitation and more variation in annual temperature. The AUC score for this model = 0.87 (± 0.05), suggesting that the model prediction was reasonable (Fig. 7A).

**Table 5.** Percentage contributions of most important bioclimatic variables to the ecological niche envelopes for all population samples of three species of *Liolaemus* described herein, plus *Liolaemus tacnae* and *Liolaemus walkeri*.

	Ancash	Ayacucho	Cusco	*Liolaemus tacnae*	*Liolaemus walkeri*
Precipitation of the Warmest Quarter	63.3			30.0	
Isotermality	31.0		28.0		
Precipitation of the Driest Quarter		64.4		21.8	
Maximum Temperature of Warmest Period		10.7			
Precipitation of the Wettest Period			55.9		43.6
Precipitation of the Wettest Quarter				12.4	
Precipitation of the Driest Period					40.6

**Table 6.** Schoener’s D values and Niche Identity test results between focal populations and species. A value in bold denotes a pair of species that has statistically distinct ENMs.

	**Ayacucho**	**Ancash**	**Cusco**	***Liolaemus tacnae***	***Liolaemus walkeri***
**Ayacucho**	1	**0.167**	**0.100**	**0.004**	**0.108**
**Ancash**		1	0.670	**0.346**	**0.300**
**Cusco**			1	**0.328**	**0.356**
***Liolaemus tacnae***				1	**0.115**
***Liolaemus walkeri***					1

The Ayacucho model did not overlap known distributions of Ancash, Cusco, *Liolaemus tacnae*, and only partially overlapped *Liolaemus walkeri* (Fig. 6C); the two most important bioclimatic variables accounting for 75.1% of the contribution to this model were Precipitation of Driest Quarter (64.4%) and Maximum Temperature of Warmest Period (10.7%; Table 5). In the permutation and jackknife tests, Precipitation of Driest Quarter was also the most important variable. In other words, the Ayacucho samples are characterized by a relatively wet and warm niche envelope, and the AUC score = 0.76 (± 0.06), suggesting that model prediction was reasonable (Fig. 7B).

The Cusco model did not overlap the known distribution of Ancash, overlapped most of Ayacuchoand *Liolaemus walkeri*, and overlapped some of *Liolaemus tacnae* (Fig. 6D). The two most important bioclimatic variables accounting for 83.9% of the contribution to the model were Precipitation of the Wettest Period and Isothermality (Table 5). In the permutation and jackknife tests, Precipitation of the Wettest Period was also the most important variable indicating a niche envelope with relative more precipitation in the wettest period of the year. The AUC score = 0.91 (± 0.03), suggesting that model prediction was reasonable (Fig. 7C).

The *Liolaemus tacnae* model did not overlap the known distributions of any of the remaining taxa (Fig. 6E); the three most important bioclimatic variables accounting for 64.2% of the contribution to the model are Precipitation of Warmest Quarter, Precipitation of Driest Quarter, and Precipitation of Wettest Quarter (Table 5). In the permutation test, the most important variable was Precipitation of the Coldest Quarter, but in the jackknife tests Precipitation of Warmest Quarter, Precipitation of Wettest Quarter and Annual Precipitation were the most important variables. This indicates that *Liolaemus tacnae* samples are characterized by a drier niche envelope relative to all other populations and the AUC score (0.85 ± 0.06) suggests that this model prediction was reasonable (Fig. 7D).

The *Liolaemus walkeri* model overlaps the known distribution of the Ancash and partially that of the Cusco samples (Fig. 6F); the two most important bioclimatic variables accounting for 84.2% of the contribution to the model are Precipitation of Driest Period and Precipitation of Wettest Period (Table 5). In the permutation and jackknife tests, Precipitation of Wettest Period also was the most important variable. This suggests a relative wetter niche envelope relative to all other populations, and the AUC score (0.77 ± 0.08) suggests that the model prediction was reasonable (Fig. 7E).

The niche identity test results showed that observed values of Schoener’s D between all populations and species were significantly lower than null distribution of pseudoreplicates except for Ancash and Cusco (Table 6).

### Integrative taxonomy

Results of mitochondrial haplotypes, binary (presence/absence of precloacal pores, spots or regular marks in lateral field, melanistic belly in adult males, ringed ventral tail pattern), morphometric (snout-vent length, axila-groin length and hindlimb length) characters and niche identity tests in various combinations, differentiated Ancash, Ayacucho and Cusco samples from each other, and from *Liolaemus tacnae* and *Liolaemus walkeri*. Despite the fact that binomial tolerance intervals showed the possible presence of polymorphisms even at a frequency cut off of 0.5% in discrete characters, we hypothesize that increasing samples sizes will lower the hypothesized frequencies of the alternative states for each taxon. Normal tolerance intervals and distributional models showed overlap between all paired combinations of samples except for the Ayacucho vs. *Liolaemus tacnae* distributional models and niche identity tests showed statistical differences between all pairwise comparisons but Ancash vs. Cusco. Note that this is an extremely conservative approach; if we simply look at the data and count the number of “fixed” differences between all combinations of samples, we would conclude that the following pairs are unambiguously diagnosed: Ayacucho, Cusco and *Liolaemus walkeri* vs. Ancash (precloacal pores or not), Ancash vs. *Liolaemus tacnae* (melanistic belly or not), Ayacucho vs. Cusco and *Liolaemus walkeri* (ringed pattern in ventral tail or not), Cusco vs. most *Liolaemus walkeri* (lateral markings or not). Based on the integration of molecular, different classes of morphological data, and niche identity test results, we conclude that *Liolaemus* populations from Ancash, Ayacucho, and Cusco can be delimited as separate species, and we describe these new species below.

## Species descriptions

### Liolaemus chavin sp. n.

http://zoobank.org/47B7926F-7D66-4C0B-9F25-9696C916E6C2

http://species-id.net/wiki/Liolaemus_chavin

Figure 8

Liolaemus alticolor Lehr 2002
Liolaemus incaicus Lobo, Quinteros and Díaz Gómez 2007
Liolaemus aff. *walkeri* Langstroth 2011

#### Holotype.

MUSM 25417, adult male collected at Conococha, Recuay Province, Ancash Department, Peru, 10.123S, 77.293W, elevation 4100 m, on 31 March 2006 by Mikael Lundberg.

#### Paratypes.

Three males (MUSM 20141, 20143, 20146) and twelve females (MUSM 25324, 25327, 25328, 25331, 25333, 25334, 25340, 25423, 25412, 30812, 30813, BYU 50192) from the same locality as the holotype. One male (MUSM 20147) from Carpa, Recuay Province, Ancash Department, on 28 February 2001 by Edgar Lehr and César Aguilar (see Data resources for elevation and coordinates). One female (MUSM 20201) from La Unión, Huánuco Department, on 3 March 1997 by Edgar Lehr (see Data resources for elevation and coordinates). Seven males (CORBIDI 10439, 10450, 10452, 10442, 10441, 10443, 10437) and six females (CORBIDI 10444, 10451, 10440, 10438, 10445, 10449) from Pampas de Huamani, San Marcos District, Huari Province, Ancash Department, on 12 February 2012 by Pablo J. Venegas (see Data resources for elevation and coordinates).

#### Diagnosis.

Small (61.7 mm maximum SVL), slender *Liolaemus* closely related to *Liolaemus walkeri*, *Liolaemus tacnae*, *Liolaemus pachacutec* sp. n. and *Liolaemus wari* sp. n. (described below) (Fig. 1). It differs from *Liolaemus walkeri*, *Liolaemus pachacutec* sp. n. and *Liolaemus wari* sp. n. in the absence of precloacal pores in males. It differs from *Liolaemus tacnae* in having a melanistic belly in adult males (not melanistic in adult *Liolaemus tacnae* males).In comparison with other species assigned to the *Liolaemus alticolor* group, *Liolaemus chavin* sp. n. differs from *Liolaemus bitaeniatus* and *Liolaemus pagaburoi* in having a smooth dorsal surface of the head (rough to slightly rough dorsal surface). It differs from *Liolaemus alticolor*, *Liolaemus aparicioi*, *Liolaemus incaicus*, *Liolaemus paulinae*, *Liolaemus pyriphlogos*, *Liolaemus puna*, and *Liolaemus variegatus* in the absence of precloacal pores in males. *Liolaemus chaltin* also lacks precloacal pores in males, but *Liolaemus chavin* sp. n. differs in having also a melanistic belly in adult males.

#### Description of holotype.

Adult male; SVL 56.8 mm; head length 13.7 mm; head width 11.3 mm; head height 7.7 mm; axilla-groin 21.0 mm (37% of SVL); foot length 10.3 mm (18.3% of SVL); tail length (regenerated) 35.2 mm (0.6 times SVL).

Fifteen dorsal head scales (from a line drawn horizontally between anterior edges of external auditory meatus to anterior border of rostral). Dorsal head scales smooth except for the interparietal and surrounding scales, scale organs more abundant in prefrontal, internasal, and supralabial regions. Five scale organs on postrostral. Nasal scale in contact with rostral, separated from first supralabial by one scale, nasal bordered by eight scales; canthus separated from nasal by one scale. Six supralabials. Six lorilabial scales, three in contact with the subocular. Six infralabials. Auditory meatus oval (height 2.3 mm, width 1.2 mm), with three small, projecting scales on anterior margin. Seven convex, smooth temporals. Orbit–auditory meatus distance 4.9 mm. Orbit–anterior margin of rostral distance 6.3 mm. Rostral almost three times wider than high (width 2.9 mm; height 1.2 mm). Mental subpentagonal, about two times as wide as high (width 3.2 mm; height 1.7 mm). Interparietal pentagonal with an elongated posterior apex, bordered by eight scales, the parietal slightly smaller. Frontal quadrangular. Supraorbital semicircles complete on both sides. Semicircles formed by 6 scales. Four enlarged supraoculars. Six distinctly imbricate superciliaries on both sides. Eleven upper and ten lower ciliaries. Subocular elongate, 3.8 mm, longer than eye diameter (2.9 mm), separated from supralabials by a single, but interrupted row of lorilabials. Second supralabial elongate, 1.9 mm. Six lorilabials with single and double rows of scale organs. Sixth, fifth and fourth lorilabials contacting subocular. Preocular small, separated from lorilabial row by one scale. Postocular as large as preocular. Mental in contact with four scales: first infralabials (on each side) and two enlarged chin shields. Chin shields forming a longitudinal row of three enlarged scales separated one from the other by seven smaller scales. Scales of throat round, flat, and imbricate. Twenty-four gulars between auditory meatus. Longitudinal neck fold without keeled scales, that are similar to dorsal in size scales. Antehumeral pocket and antehumeral neck fold well developed. Forty-two scales between auditory meatus and shoulder (counting along postauricular and longitudinal neck fold), thirty-two scales between auditory meatus and antehumeral neck fold. Gular folds absent.

Dorsal scales rhomboidal, keeled, and imbricate. Sixty-six dorsal scales between occiput and level of groin. Sixty-two scales around midbody. Thirty rows of keeled scales on dorsum at midtrunk. Scales become smooth along flank and toward belly. Ventral scales slightly wider than dorsals. Eighty-two ventral scales between mental and cloaca; no precloacal pores. Supracarpals laminar, round, and smooth. Subdigital lamellae of fingers with three keels, in number I: 6; II: 11; III: 14; IV: 15; V: 10 (right hand). Claws moderately long. Supradigital lamellae convex, smooth, and imbricate. Infracarpals and infratarsals keeled, distinctly imbricate. Supratarsals smooth. Subdigital lamellae of toes I: 13; II: 13; III: 13; IV: 12; V: 6 (right foot).

#### Color pattern in preservation.

Dorsal background color from occiput to base of tail greenish brown. Black continuous vertebral stripe present. Dark paravertebral marks. Paravertebral and vertebral fields of same background color. Dorsolateral stripes distinctly cream-color. Small dark cream-colored markings scattered in lateral field. Cream ventrolateral stripe, beginning on the upper auricular meatus, continuing across the longitudinal neck fold, through the shoulders, ending in the groin. Dark and small cream-colored marks in the ventral field. Black ventral color from about second third of head to femur, tibia and first third of tail. Dark and cream-colored small markings in first third of ventral head and two posterior thirds of tail.

#### Color pattern in life.

Head dorsally brown with black and light brown dots. Subocular cream colored, dorsum bisected by a dark vertebral line. Vertebral field not conspicuous, bordering the vertebral line with a tenuous yellowish line. Paravertebral field with dark marks, bordered dorsally by a yellowish cream dorsolateral stripe. Lateral field with black and yellow reticulated pattern and white dots. Inconspicuous ventrolateral stripe, beginning on upper margin of auricular meatus, continuing from the longitudinal neck fold, through the shoulders, ending in the groin. Ventrolateral similar to lateral field but with more white dots. Fore and hind limbs same color as the paravertebral field, with diffuse dorsal markings. Dark, melanistic ventral color from about second third of head to femur, tibia and first third of tail. Dark and white dots in first third of ventral head and two posterior thirds of tail.

#### Variation.

Variation in characters is summarized in Tables 1–4. There is sexual dichromatism. Adult males exhibit melanistic belly, cloacal region and throat, or melanistic belly only; adult females exhibit black and white spots on belly, cloacal region and throat, or yellowish belly and tail.

#### Etymology.

The specific epithet *chavin* refers to the pre-Inca culture Chavin, which had its center close to the type locality and frequently depicted reptile figures on some of its most remarkable sculptures. The species name is in the nominative singular.

#### Distribution and natural history.

*Liolaemus chavin* sp. n. is known from four localities in the central Andes, at elevations of 3535–4450 m in Ancash and Huánuco Departments in western central Peru (Fig. 11). It is the northernmost species of the subgenus *Liolaemus*.

**Figure 8.**
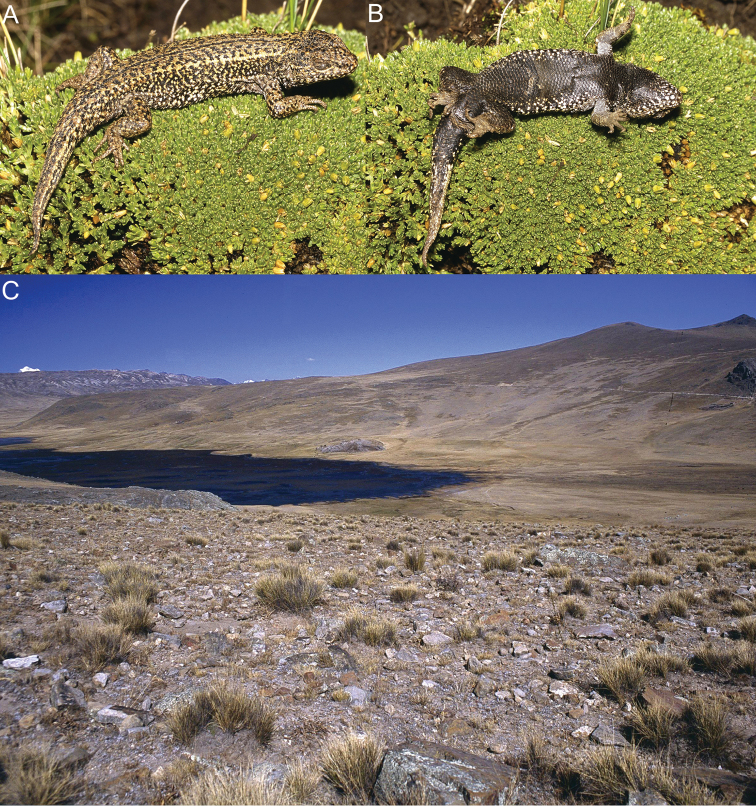
Dorsal (**A**) and ventral (**B**) views of the holotype of *Liolaemus chavin* sp. n. (**C**) Type locality.

*Liolaemus chavin* sp. n. was found active and under rocks in grassland and shrubland habitats at higher and lower elevations respectively (Fig. 8). In Pampas de Huamani the new species was usually found basking on grass up to 60 cm above the ground, and when they were disturbed they escaped into the base of grass clumps. Individuals basking on rocks were very rare in all localities. On cloudy days we found this species inactive hidden in the base of grass clumps, although some individuals were also found inactive under rocks. This species is viviparous; one female showed two uterine chambers per side with developed embryos, yolk and no visible shell in either chamber, and three females showed two uterine chambers per side with yolk, without developed embryos and no visible shell in each chamber. At the type locality no sympatric species of reptiles were found, but four amphibians are known: *Pleurodema marmoratum* (Duméril & Bibron, 1840), *Telmatobius mayoloi* Salas & Sinsch, 1996, *Gastrotheca peruana* and *Rhinella (Bufo) spinulosa* (Wiegmann, 1834) (Lehr 2002; personal observations). Sympatric species at Catac include the anurans *Gastrotheca peruana*, *Rhinella (Bufo) spinulosa*, *Telmatobius rimac* Schmidt, 1954, *Telmatobius mayoloi*, and the lizard *Stenocercus chrysopygus* Boulenger, 1900; at Carpa, *Gastrotheca peruana* (Boulenger, 1900), *Rhinella (Bufo) spinulosa* and *Pleurodema marmoratum*; at Pampas de Huamani, *Gastrotheca peruana*, *Pleurodema marmoratum* and *Rhinella (Bufo) spinulosa*; and at La Unión, *Gastrotheca griswoldi* Shreve, 1941, *Gastrotheca peruana*, *Rhinella (Bufo) spinulosa* and *Stenocercus chrysopygus* (Lehr, 2002).

### Liolaemus pachacutec sp. n.

http://zoobank.org/A979BB00-3CA1-47C9-8EB0-F605166FBF1A

http://species-id.net/wiki/Liolaemus_pachacutec

Figure 9

#### Holotype.

MUSM 29683, adult male collected at Challabamba, Paucartambo Province, Cusco Department, Peru, 13.254S, 71.838W, elevation 4364 m, on 1 April 2009 by César Ramírez.

#### Paratypes.

Three males (MUSM 29681, 29687, 29678) and four females (MUSM 29679, 29689, 29680, 29682) from the same locality as the holotype. Two males MUSM (29665, 29668) and one female (MUSM 29669) from Lamay, Calca Province, Cusco Department, on 12 October 2009 by César Ramírez (see Data resources for elevations and coordinates). One male (MUSM 29664), two females (MUSM 29688, BYU 50237) and one juvenile (MUSM 31412) from Pisac, Calca Province, Cusco Department, on 4 July and 11 October 2009 by César Ramírez, and on 28 June 2012 by César Aguilar, Perry Wood and Juan Carlos Cusi (see Data resources for elevations and coordinates). One male (MUSM 31540), two females (MUSM 31538-39) and one juvenile (MUSM 31537) from Tiaparo, Pocohuanca District, Aymaraes Province, Apurímac Department, on 11 June 2013 by Alfredo Guzmán (see Data resources for elevations and coordinates).

#### Diagnosis.

Small (51.9 mm maximum SVL) *Liolaemus* closely related to *Liolaemus chavin* sp. n., *Liolaemus tacnae*, *Liolaemus walkeri*, and *Liolaemus wari* sp. n. (described below) (Fig. 1). It differs from *Liolaemus chavin* sp. n. and *Liolaemus tacnae* in having precloacal pores (males). *Liolaemus pachacutec* differs from *Liolaemus wari* sp. n. in having a partial or complete melanistic belly in adult males and in lacking a ringed pattern in ventral tail. *Liolaemus pachacutec* differs from most individuals (90%) of *Liolaemus walkeri* in lacking spots in the lateral field. In comparison with other species assigned to the *Liolaemus alticolor* group, *Liolaemus pachacutec* differs from *Liolaemus chaltin* in having precloacal pores in males. It differs from *Liolaemus paulinae* in the presence of a vertebral line and smooth neck scales. It differs from *Liolaemus puna*, *Liolaemus alticolor* and *Liolaemus incaicus* in having a partial or complete melanistic belly in adult males. It differs from *Liolaemus aparicioi* in lacking keeled temporal scales. It differs from *Liolaemus bitaeniatus* and *Liolaemus pagaburoi* in having a smooth dorsal surface of the head. It differs from *Liolaemus pyriphlogos* in the absence of red marks in lateral fields. It differs from *Liolaemus variegatus* in lacking keeled temporal scales, rugose dorsal head scales, and precloacal pores in females.

#### Description of holotype.

Adult male; SVL 44.8 mm; head length 11.0 mm; head width 8.2 mm; head height 6.2 mm; axilla-groin distance 18.4 mm (41.1% of SVL); foot length 13.6 mm (30.4% of SVL); tail length 74.9 mm. (1.7 times SVL).

Dorsal head scales 16, dorsal head scales smooth, scale organs more abundant in loreal and supralabial regions. Two scale organs on postrostral. Nasal scale in contact with rostral, separated from first supralabial by one scale, nasal bordered by six scales; canthus separated from nasal by one scale. Four supralabials. Four lorilabials scales and one in contact with the subocular. Five infralabials. Auditory meatus oval (height 2.0 mm, width 1.0 mm), with two small, projecting scales on anterior margin. Six convex, smooth temporals (counting vertically from buccal commissure to posterior corner of orbit). Orbit–auditory meatus distance 3.9 mm. Orbit–anterior margin of rostral distance 4.3 mm. Rostral about two times wider than high (width 2.3 mm; height 1.0 mm). Mental subpentagonal, about two times as wide as high (width 2.5 mm; height 1.0 mm). Interparietal pentagonal with an elongated posterior apex, bordered by five scales, the parietal of similar size. Frontal trapezoidal.

Supraorbital semicircles complete on both sides. Semicircles formed by six scales. Five enlarged supraoculars. Six distinctly imbricate superciliaries on both sides. Eleven upper and lower ciliaries. Subocular elongate, 2.8 mm, longer than eye diameter (2.1 mm; measured between anterior and posterior commissure of ciliaries), separated from supralabials by a single, but interrupted row of lorilabials. Fourth supralabial elongate, 2.0 mm. Four lorilabials with single row of scale organs. Fourth lorilabial contacting subocular. Preocular small, separated from lorilabial row by one scale. Postocular as large as preocular. Mental in contact with four scales: first infralabials (on each side) and two enlarged chin shields. Chin shields forming a longitudinal row of four enlarged scales separated one from the other by six smaller scales. Scales of throat round, flat, and imbricate. Twenty-two gulars between auditory meatus. Longitudinal neck fold without keeled scales and smaller in size than dorsal scales. Antehumeral pocket and antehumeral neck fold well developed. Thirty-six scales between auditory meatus and shoulder (counting along postauricular and longitudinal neck fold), twenty-six scales between auditory meatus and antehumeral neck fold. Gular folds absent.

Dorsal scales rhomboidal, keeled, and imbricate. Forty-two dorsal scales between occiput and level of groin. Forty-five scales around midbody. Nineteen rows of keeled scales on dorsum at midtrunk. Scales becoming smooth along flank and toward belly. Ventral scales slightly wider than dorsals. Seventy-seven ventral scales between mental and precloacal pores. Five precloacal pores. Supracarpals laminar, round, and smooth. Subdigital lamellae of fingers with three keels, in number I: 8; II: 12; III: 16; IV: 18; V: 12 (right fingers). Claws moderately long. Supradigital lamellae convex, smooth, and imbricate. Infracarpals and infratarsals keeled, distinctly imbricate. Supratarsals smooth. Subdigital lamellae of toes I: 10; II: 14; III: 18; IV: 22; V: 15 (right toes).

#### Color in preservation.

Dorsal background color from occiput to base of tail brownish-green. Black thin continuous vertebral line present. No dark paravertebral marks. Paravertebral and vertebral fields with same background color. Distinct cream dorsalateral stripes. No marks in lateral field. Cream ventrolateral stripes, beginning on the posterior corner of the eye, continuing across the upper auricular meatus, the longitudinal neck fold, through the shoulders, ending in the groin. No marks in the ventral field. Melanistic venter on throat, femur, tibia, and belly. Small and scattered dark marks in chin area and ventrolateraly. Ventral tail melanistic near the cloaca, with a thin longitudinal stripe, first half with small marks lateral to the stripe.

#### Color pattern in life.

Head dorsally brown with scattered black dots. Subocular white. Thin and faint black vertebral line. Paravertebral field without dark marks. Creamy dorsolateral stripes. Lateral field without marks. Faint cream-white ventrolateral stripe, beginning on upper margin of eye, continuing from auricular meatus, the longitudinal neck fold, through the shoulders, ending in the groin. Ventral field yellow. Forelimbs and chin scales white with scattered black dots. Melanistic belly, hind limbs, posterior two thirds of throat. Belly with scattered yellow dots laterally. Tail with a black region close to the cloaca, black longitudinal stripe and dots at each side of the stripe.

#### Variation.

Variation in characters is summarized in Table 1–4. There is sexual dichromatism. Males have a complete or partial melanistic belly and throat, while females have a white or yellow belly and black spots on throat. Some males have orange and yellow dots on lateral belly and yellow dots on chin scales, and ventral field with orange and black dots.

#### Etymology.

The specific epithet *pachacutec* refers to one of most important Inca rulers, Pachacutec, who built the best known Inca ruins, including Machu Picchu and Pisac, this last site at a higher elevation just above the type locality. The species name is in the nominative singular.

#### Distribution and natural history.

**Figure 9.**
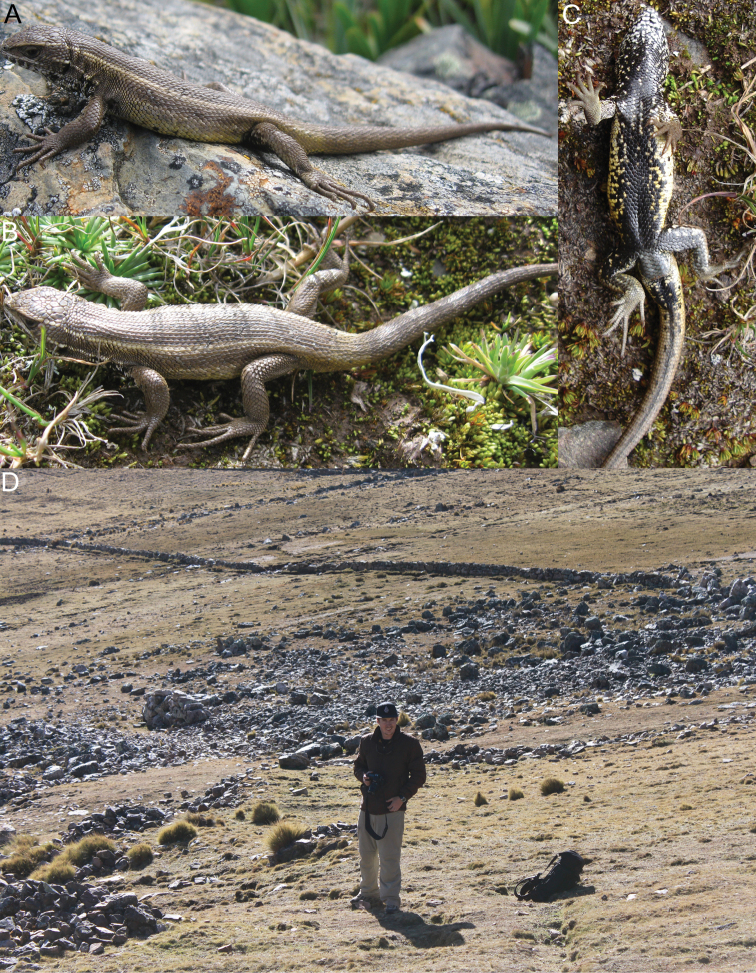
Lateral (**A**) dorsal (**B**) and ventral (**C**) views of the holotype of *Liolaemus pachacutec* sp. n. (**D**) Habitat of *Liolaemus pachacutec*

*Liolaemus pachacutec* sp. n. is known from four localities in the central Andes, at elevations of 4023–4972 m in the departments of Cusco and Apurímac in southeastern Peru (Fig. 11). The species was found under rocks in grassland habitats (Fig. 9). It was found in sympatry at similar elevations with *Liolaemus ortizi* Laurent, 1982 and *Tachymenis peruviana* Wiegmann, 1835. This species is probably viviparous; two females showed one or two uterine chambers per side, with an embryo and abundant yolk in each chamber, but without a visible shell.

### Liolaemus wari sp. n.

http://zoobank.org/67A997B8-5854-4D0D-B1E0-77680FF47512

http://species-id.net/wiki/Liolaemus_wari

Figure 10

Liolaemus walkeri Lobo and Espinoza 1999
Liolaemus walkeri Martínez Oliver and Lobo 2002
Liolaemus walkeri Lobo, Quinteros and Díaz Gómez 2007
Liolaemus walkeri Quinteros 2012
Liolaemus walkeri Ocampo, Aguilar-Kirigin and Quinteros 2012

#### Holotype.

MUSM 30837, adult male collected at Abra Toccto, Huamanga Province, Ayacucho Department, Peru, 13.345S, 74.167W, elevation 4231 m, on 4 June 2012 by César Aguilar and Víctor Vargas.

#### Paratypes.

Three males (MUSM 30823, BYU 50184, 50185) and ten females (MUSM 30824, 30825, 30826, 30827, 30828, 30831, BYU 50186, 50187, 50191, 50243) from the same locality as the holotype. Two males (MUSM 30830, 30834) and three females (MUSM 30829, BYU 50188, 50190) from high area above the Historic Sanctuary Pampas, Huamanga Province, Ayacucho Department, on 3 June 2012 by César Aguilar and Víctor Vargas (see Data resources for elevations and coordinates). Two males (MUSM 25703, 25704) and one female (MUSM 25702) from Yanacocha Lake, La Mar Province, Ayacucho Department, on 24 November 2010 by Margarita Medina (see Data resources for elevations and coordinates). Two females (MUSM 25719, BYU 50189) from Huaychao, Huamanga Province, Ayacucho Department, on 1 December 2010 by Margarita Medina (see Data resources for elevations and coordinates). Two females (MUSM 30243, 30244) from Tambo, San Miguel Province, Ayacucho Department, by Michael Harvey. One male (MUSM 31411) and two juveniles (BYU 50235-36) from about 45 Km west Puquio-Cusco roadway, Lucanas Province, Ayacucho Department, on 11 June 2012 by César Aguilar and Víctor Vargas (see Data resources for elevations and coordinates).

#### Diagnosis.

Small (61.4 mm maximum SVL), slender *Liolaemus*, closely related to *Liolaemus chavin* sp. n., *Liolaemus pachacutec* sp. n., *Liolaemus tacnae* and *Liolaemus walkeri* (Fig. 1). It differs from *Liolaemus chavin* sp. n., *Liolaemus pachacutec* sp. n. and *Liolaemus walkeri* in having a ringed pattern on the ventral tail of adult males. It differs from *Liolaemus pachacutec* sp. n. in having spots in the lateral fields. *Liolaemus wari* differs from *Liolaemus tacnae* and *Liolaemus chavin* in having precloacal pores in males. In comparison with other species assigned to the *Liolaemus alticolor* group, *Liolaemus wari* sp. n. differs from *Liolaemus chaltin* in having precloacal pores in males. It differs from *Liolaemus paulinae* in lacking keeled neck scales. It differs from *Liolaemus puna*, *Liolaemus alticolor* and *Liolaemus incaicus* in having black spots on belly of adult males. It differs from *Liolaemus aparicioi* in lacking keeled temporal scales. It differs from *Liolaemus bitaeniatus* and *Liolaemus pagaburoi* in having a smooth dorsal surface of the head (rough to slightly dorsal surface of the head). It differs from *Liolaemus pyriphlogos* in the absence of red marks in the lateral field (red marks in the lateral fields present). It differs from *Liolaemus variegatus* in the absence of keeled temporal scales, rugose dorsal head scales and precloacal pores in females.

#### Description of holotype.

Adult male; SVL 55.4 mm; head length 11.4 mm; head width 9.8 mm; head height 6.2 mm; axilla–groin distance 23.3 mm (42% of SVL); foot length 15.0 mm. (27.1% of SVL); tail length 83.7 mm. (1.5 times SVL).

Dorsal head scales 14, dorsal head scales smooth, scale organs more abundant in loreal and supralabial regions. Five scale organs on postrostral. Nasal scale in contact with rostral, separated from first supralabial by one scale, nasal bordered by seven scales; canthus separated from nasal by one scale. Four supralabials. Five lorilabials scales and two in contact with the subocular. Four infralabials. Auditory meatus oval (height 2.0 mm, width 1.9 mm), with two small, projecting scales on anterior margin. Seven convex, smooth temporals (counting vertically from buccal commissure to posterior corner of orbit). Orbit–auditory meatus distance 4.6 mm. Orbit–anterior margin of rostral distance 7.9 mm. Rostral almost three times wider than high (width 2.7 mm; height 1.0 mm). Mental subpentagonal, about two times as wide as high (width 2.6 mm; height 1.2 mm). Interparietal pentagonal with an elongated posterior apex, bordered by seven scales, the parietal slightly smaller. Frontal trapezoidal. Supraorbital semicircles complete on both sides. Semicircles formed by 6 scales. Four enlarged supraoculars. Five distinctly imbricate superciliaries on both sides. Eleven upper and lower ciliaries. Subocular elongate, 3.2 mm, longer than eye diameter (2.3 mm; measured between anterior and posterior commissure of ciliaries), separated from supralabials by a single, but interrupted row of lorilabials. Second supralabial elongate, 1.6 mm. Five lorilabials with single and double rows of scale organs. Fifth and fourth lorilabials contacting subocular. Preocular small, separated from lorilabial row by one scale. Postocular as large as preocular. Mental in contact with four scales: first infralabials (on each side) and two enlarged chin shields. Chin shields forming a longitudinal row of three enlarged scales separated one from the other by six smaller scales. Scales of throat round, flat, and imbricate. Twenty-one gulars between auditory meatus. Longitudinal neck fold without keeled scales and smaller in size than dorsal scales. Antehumeral pocket and antehumeral neck fold well developed. Twenty-nine scales between auditory meatus and shoulder (counting along postauricular and longitudinal neck fold), 21 scales between auditory meatus and antehumeral neck fold. Gular folds absent.

Dorsal scales rhomboidal, keeled, and imbricate. Forty-four dorsal scales between occiput and level of groin. Fifty-three scales around midbody. Twenty-two rows of keeled scales on dorsum at midtrunk. Scales becoming smooth along flank and toward belly. Ventral scales slightly wider than dorsals. Seventy-three ventral scales between mental and precloacal pores. Five precloacal pores. Supracarpals laminar, round, and smooth. Subdigital lamellae of fingers with three keels, in number I: 8; II: 12; III: 16; IV: 16; V: 10 (right fingers). Claws moderately long. Supradigital lamellae convex, smooth, and imbricate. Infracarpals and infratarsals keeled, distinctly imbricate. Supratarsals smooth. Subdigital lamellae of toes I: 8; II: 12; III: 16; IV: 20; V: 13 (left toes).

#### Color pattern in preservation.

Dorsal background color from occiput to base of tail brownish-green. Black continuous vertebral line present. Dark paravertebral marks. Paravertebral and vertebral fields with same background color. Highly distinct creamy-yellow dorsalateral stripes. Large dark and small cream marks in lateral field. Cream ventrolateral stripe, beginning on the posterior corner of the eye, continuing across the upper auricular meatus, the longitudinal neck fold, through the shoulders, ending in the groin. Dark and cream small marks in the ventral field. Black spots on throat, femur, tibia, posterior third of belly and laterally in anterior two thirds of belly. Small and scattered dark marks in chest and anterior two thirds of belly. Tail with dark horizontal rows.

#### Color pattern in life.

Head dorsally brown with black dots. Subocular cream. A black vertebral band with a thin yellow stripe on the middle. The vertebral band has a thin white stripe on each side. Paravertebral field with dark marks with posterior white dots. Creamy-yellow dorsolateral stripes. Lateral field with black marks separated by cream diagonal stripes. Yellowhish-white ventrolateral stripe, beginning on upper margin of eye, continuing from auricular meatus, the longitudinal neck fold, through the shoulders, ending in the groin. Ventrolateral similar to lateral field and same color as the paravertebral field, with diffuse dorsal markings. Forelimbs, chest and belly yellowish-white with scattered and diffuse black dots. Black marks on hind limbs, throat, and posterior third of belly. Tail with black horizontal bands separated by white bands.

#### Variation.

The variation in morphological characters is shown in Tables 1–4. There is sexual dichromatism. Males have white or yellow belly and throat covered completely with black spots, yellowish belly and throat with black spots on posterior third of belly, or a melanistic belly on posterior third and cloacal region, with black dots on a white throat; females have white belly and yellowish throat with faint black dots, yellowish belly and throat with faint black spots, or yellowish belly and throat without spots. Adult males have white, yellowish and yellow tails with a conspicuous ringed pattern; adult females have white, yellowish or reddish ventral tails with or without a faint ringed pattern.

#### Etymology.

The specific epithet *wari* refers to the pre-Inca culture Wari (600–850 AD), which had its center close to the type locality. The species name is in the nominative singular.

#### Distribution and natural history.

*Liolaemus wari* sp. n. is known from seven localities in the central Andes, at elevations of 3768–4246 m in Ayacucho Department in eastern southern Peru (Fig. 11).

**Figure 10.**
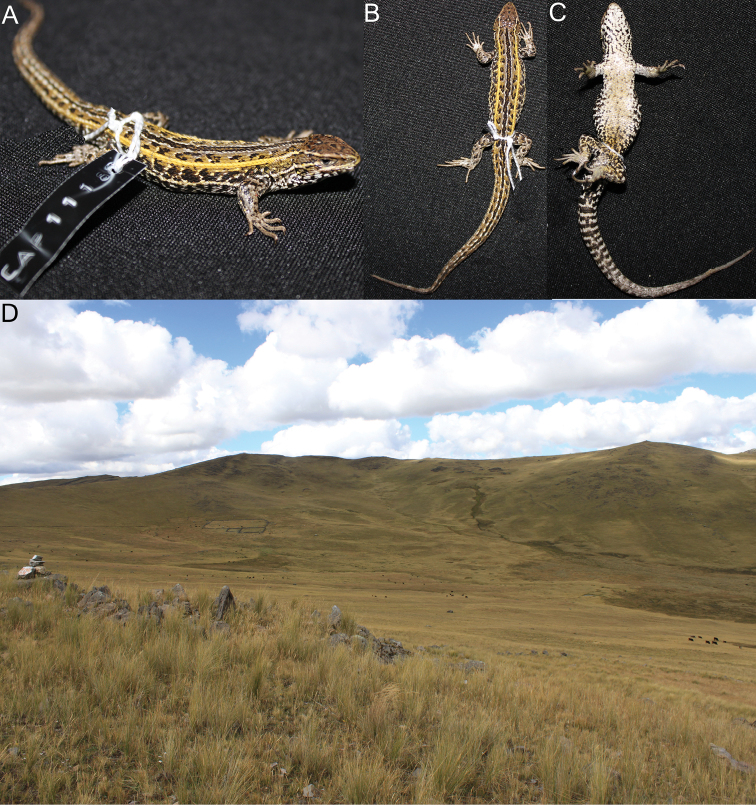
Lateral (**A**) dorsal (**B**) and ventral (**C**) views of the holotype of *Liolaemus wari* sp. n. (**D**) Type locality.

*Liolaemus wari* sp. n. was active on the ground or found under rocks in grassland (Fig. 10) and shrubland habitats. It was found in sympatry with another *Liolaemus* species belonging to the *Liolaemus montanus* series and the snake *Tachymenis peruviana*. This species is probably viviparous; three females each showed three uterine chambers per side; each chamber showed yolk, but with no developed embryos or visible shell.

**Figure 11.**
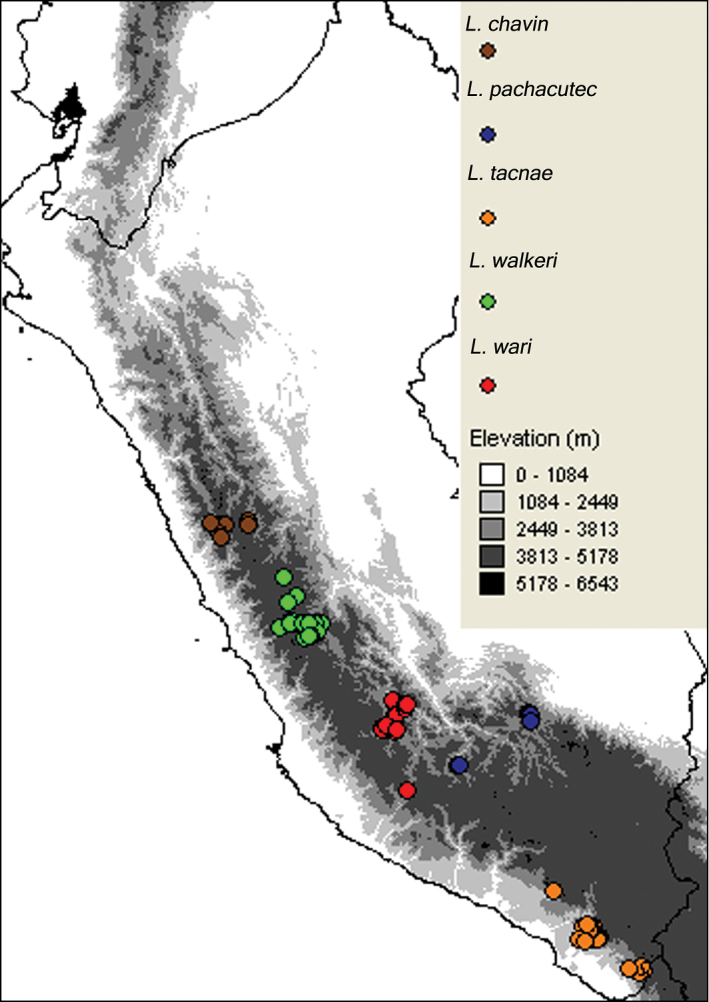
Geographic distribution of *Liolaemus chavin*, *Liolaemus pachacutec*, *Liolaemus tacnae*, *Liolaemus walkeri*, and *Liolaemus wari*.

## Discussion

### Phylogenetic relationships

Surprisingly, our phylogenetic analysis showed that the three new species described herein plus *Liolaemus tacnae* and *Liolaemus walkeri*, assigned to *alticolor-bibronii* group, are strongly separated from the other members of this species group included in this study. Specifically, the species *Liolaemus alticolor* and *Liolaemus incaicus* assigned to the *alticolor-bibronii* group (Lobo et al. 2010, Quinteros 2013) were not recovered with *Liolaemus tacnae*, *Liolaemus walkeri*, and the three new species.

Previous molecular based phylogenies did not include *Liolaemus alticolor*, *Liolaemus tacnae* and/or *Liolaemus walkeri* (Espinoza et al. 2004, Morando et al. 2007, Schulte and Moreno-Roark 2010) and much of what these different topologies show (including ours) is probably an artifact of incomplete taxon/population sampling. Previous morphology-based phylogenies included better taxon sampling, but all of them recovered clades with low or no statistical support, and relationships of *Liolaemus tacnae*, *Liolaemus walkeri*, *Liolaemus alticolor*, and *Liolaemus incaicus* with each other and other species assigned to the *alticolor-bibronii* groupare ambiguous.

### Species delimitation and integrative taxonomy

We take our results based the mtDNA gene tree as a first step in species “discovery” (Carstens et al. 2013), and identify the Ancash, Ayacucho, and Cusco clades as “candidate species” (Morando et al. 2003, Avila et al. 2004). Comparative morphological and niche envelope assessments of these three clades revealed combinations of characters from three different lines of evidence, that unambiguously diagnose these groups as distinct from each other and from *Liolaemus tacnae* and *Liolaemus walkeri* (this is the second step of species delimitation – “validation” – following Carstens et al. 2013). This result highlights the need for using an integrative approach rather than a single line of evidence (e.g. morphology, usually meristic data only) to delimit species.

Our results show that normal tolerance intervals of continuous morphometric and meristic characters could not discriminate between any of these new species nor between *Liolaemus tacnae* and *Liolaemus walkeri*. On the other hand, discrete character analysis revealed some diagnostic characters, including: (1) the presence/absence of pre-cloacal pores in males distinguishing *Liolaemus chavin* and *Liolaemus tacnae* from *Liolaemus pachacutec*, *Liolaemus walkeri*, and *Liolaemus wari*; (2) the presence/absence of a complete or partial melanistic belly in adult males distinguishing *Liolaemus chavin* from *Liolaemus tacnae*; (3) the presence/absence of a ringed ventral tail pattern of adult males distinguishing *Liolaemus wari* from *Liolaemus pachacutec* and *Liolaemus walkeri*; and (4) the presence/absence of regular marks or spots in lateral fields distinguishing *Liolaemus pachacutec* from *Liolaemus wari* and from most (90%) individuals of *Liolaemus walkeri*. However, binomial tolerance intervals showed that all these “fixed” character states in our samples have a high probably of non-fixation when statistical inference is extended to consider large sample sizes. Despite these findings, we encourage the use of these binomial tests to place empirical evidence into a broader context, and to make investigators aware that tolerance intervals will become narrower as sample sizes increase, and that taxonomic decisions should be based on statistical populations not on samples (Zapata and Jiménez 2012). Moreover, samples taken at random are important for strong statistical inferences, but obtaining random samples in observational studies (such as in most taxonomic studies) is often impractical or impossible, and thus potential for bias is a serious concern (Ramsey and Schafer 2002). Besides this limitation, a statistical inference (such as those based on tolerance intervals) is better than no inference at all. However, statistic tests that evaluate differences in central tendencies (e.g., the ANOVA and Mann-Whitney *U* tests we used here) do not seem relevant as SDL criteria or for practical taxonomic purposes. For instance, most pairwise comparisons of SVL between focal populations (Ancash, Ayacucho, Cusco) and species (*Liolaemus tacnae* and *Liolaemus walkeri*) are significant in an ANOVA test at a confidence level of 0.05, giving the false impression that this character is useful for species delimitation or taxonomic identification, but tolerance intervals indicate that these populations and species completely overlap with respect to this character (Table 2).

Molecular analysis and, in most cases, niche identity tests, support our species units based on these few morphological characters, and in combination provide more robust hypotheses. Our model-based molecular phylogenetic analysis provided the basis for our “candidate species” hypotheses, but molecular phylogenetic analysis relies on the assumption that a chosen evolutionary model is a correct one (Posada 2009), and we recognize that in the absence of corroboration from independent data sets, mtDNA may often over-split species (Miralles and Vences 2013). However, assumptions are also pervasive in morphological and ENM analyses. Discovery of gaps in morphology assumes that discontinuities are not due polymorphisms, ontogenetic variation or phenotypic plasticity (Wiens and Servedio 2000, Zapata and Jiménez 2012), and ENM (especially those models based on background data and not true absence records) assumes that occupied distribution of a species is not reduced by biotic interactions and dispersal limitations (Peterson et al. 2011). Despite these assumptions, we think that robust hypotheses of species delimitation based on different data sets give stability to scientific names, provide the strongest inference about species boundaries, overcome overlapping character variation in any particular character system, and should be a prioritized research theme in systematics (Balakrishnan 2005, Will et al. 2005, Padial and De la Riva 2006, Padial et al. 2010). In addition, we expect more exciting results when new molecular coalescent-based multi-locus and morphological multivariate methods can be applied to our data (Zapata and Jiménez 2012, Camargo and Sites 2013).

### Northern limits of squamate viviparity in the high Andes

*Liolaemus chavin* is the northernmost viviparous species of the subgenus *Liolaemus*. Two recognized *Liolaemus* species present in the extreme northern range of the genus are *Liolaemus robustus* Laurent, 1992 and *Liolaemus disjunctus* Laurent, 1990 (subgenus *Eulaemus*). In the case of *Liolaemus disjunctus*, our recent fieldwork in the area of the species’ type locality did not locate any specimen. The same result was found when we revisited localities near the type locality of *Liolaemus disjunctus* in 2012, and to our knowledge this species has not been collected at least since its original description and data on its reproductive mode are still lacking (Laurent 1990). On the other hand, the colubrid snake *Tachymenis peruviana* is another viviparous squamate widely distributed in the high Andes of Argentina, Bolivia, Chile and Peru. Its northern limits are in the department of La Libertad, Peru at about latitude 7°S, and no other viviparous squamate species are present in the high Andes of northernmost Peru, Ecuador, and Colombia.

What selective pressures might have limited the distribution of viviparous squamates in the high Andes? Although there are no field or experimental studies that have addressed this question in particular, one distributional pattern seems to be evident in the northern distributional limit of *Liolaemus*. For instance, on the Pacific Andean slopes at about latitude 15°S and south in Peru, viviparous *Liolaemus* species are present in lower, middle and higher elevations (C. Aguilar, personal observations), and oviparous lizards (genera *Phyllodactylus*, *Ctenoblepharys* and *Microlophus* but not *Stenocercus*) are only present at lower and middle elevations. However, on the Pacific Andean slopes at about latitude 12°S, *Liolaemus* species are only present at higher elevations and oviparous *Stenocercus* (Tropiduridae) species become common at lower and middle elevations, together with the above-mentioned oviparous genera. If we consider the actual northern limits of *Liolaemus* as represented by *Liolaemus chavin*, viviparous lizards in the high Andes do not extend north beyond about latitude 8–9°S. North of latitude 8°S, oviparous *Stenocercus*, *Petracola* and *Riama* (Gymnophthalmidae) species are the only lizard genera present in the high Andes of Peru and Ecuador. One interesting distributional and reproductive pattern that matches this change in reproductive mode in lizards is the distribution pattern of amphibians with direct development (genus *Pristimantis*). No *Pristimantis* species have been found in sympatry with northernmost *Liolaemus* species. At high elevations on the Pacific slopes, the northernmost *Liolaemus* species (*Liolaemus chavin* and *Liolaemus robustus*) have always been found with anurans having complete (genera *Rhinella*, *Pleurodema* and *Telmatobius*) or partial (*Gastrotheca*) indirect development.

Direct-development *Pristimantis* rely on high humidity substrates for egg development (Duellman and Lehr 2009), and what may have limited the distribution of direct-development frogs in the Pacific basin of southern Peru and northern Chile, and the Andean Plateau, is the formation of an Arid Diagonal area due to the interaction of the Humboldt Current and uplift of the Andes. If so, then a working hypothesis for the evolution of viviparity and placentation in some clades of *Liolaemus* is their relationship to the presence of these arid and hypoxic conditions. Arid environments in hypoxic middle and high elevations might be lethal to the development of oviparous lizard eggs. However, origins of viviparity in *Liolaemus* seem to be associated with shifts to cold climates (e.g., in the Oligocene; Schulte and Moreno-Roark 2010), thus supporting the cold climate hypothesis (CCH; Tinkle and Gibbons 1977). According to this hypothesis, viviparity has evolved to avoid lethal ambient temperatures in high elevations and latitudes, and through retention of eggs in the uterus coupled with female behavioral thermoregulation, this mode accelerates embryonic development (for a recent review see Sites et al. 2011). The CCH is a special case of a more general maternal manipulation hypothesis (MMH) where females can enhance fitness-related phenotypic attributes in offspring by manipulating thermal conditions during embryogenesis (Shine 1995). However, arid environments may be more important with increasing hypoxic conditions in high altitudes for the evolution of viviparity than cold climates, as has been suggested for *Phrynosoma* lizards (Hodges 2004, but see Lambert and Wiens 2013). In other words, altitude may be a surrogate of other selective factors important for the evolution of viviparity, not only cold climates (Hodges 2004). High altitude environments tend to be drier and have low oxygen conditions, and viviparous species may be able to provide a better oxygen environment for developing embryos via placental structures (Hodges 2004). Whether shifts in cold climates and/or appearance of arid zones along with Andean uplift are correlated with the origin of viviparity in *Liolaemus* should be tested with coalescent based multi-locus phylogenetic studies and a time-calibrated hypothesis of species relationships.

### Key to Peruvian species of the subgenus *Liolaemus*

**Table:** 

1a	Dorsal body with mucronated scales, no melanistic or without black spots on throat or belly in males	*Liolaemus alticolor* group
1b	Dorsal body usually without mucronated scales, melanistic or with spots on throat or belly in males	3
2a	Dorsal pattern without spots	*Liolaemus alticolor*
2b	Dorsal pattern with spots	*Liolaemus incaicus*
3a	Males without precloacal pores	4
3b	Males with precloacal pores	5
4a	Males with black spots on throat, no melanistic belly	*Liolaemus tacnae*
4b	Males with melanistic belly	*Liolaemus chavin*
5a	Males with ringed pattern in ventral tail, mucronated scales present or absent	*Liolaemus wari*
5b	Males without ringed pattern in ventral tail, mucronated scales absent	6
6a	Spots absent in the lateral fields	*Liolaemus pachacutec*
6b	Spots present in the lateral fields (most individuals)	*Liolaemus walkeri*

## Supplementary Material

XML Treatment for Liolaemus chavin

XML Treatment for Liolaemus pachacutec

XML Treatment for Liolaemus wari

## Appendix 1

Supplementary file 1. (doi: 10.3897/zookeys.364.6109.app1) File format: Microsoft Excel (xls).

**Explanation note:** GenBank accesion and museum voucher numbers of haplotypes used in this study.

## Appendix 2

Supplementary file 2. (doi: 10.3897/zookeys.364.6109.app2) File format: Microsoft Excel (xls).

**Explanation note:** Museum voucher data of specimens used in the morphological analyses.

## Appendix 3

Supplementary file 3. (doi: 10.3897/zookeys.364.6109.app3) File format: Microsoft Excel (xls).

**Explanation note:** Occurrence records and locality information of *Liolaemus tacnae*, *Liolaemus walkeri* and the three new species described in this study, and used to develop ecological niche models.

## Appendix 4

Supplementary file 4. (doi: 10.3897/zookeys.364.6109.app4) File format: Microsoft Excel (xls).

**Explanation note:** Eigenvalues, percentage of variance and similarity accounted by principal components and correspondence axes 1 and 2 respectively.
